# Association of Cervical Dysbacteriosis, HPV Oncogene Expression, and Cervical Lesion Progression

**DOI:** 10.1128/spectrum.00151-22

**Published:** 2022-08-29

**Authors:** Hang Liu, Hua Liang, Di Li, Ming Wang, Yan Li

**Affiliations:** a Department of Clinical Laboratory, Renmin Hospital of Wuhan University, Wuhan, China; b Department of Obstetrics and Gynecology, Renmin Hospital of Wuhan University, Wuhan, China; University of Arizona

**Keywords:** cervical cancer, HPV oncogene, vaginal microbiome, metagenomic sequence

## Abstract

As the fourth most common gynecological cancer, cervical cancer has resulted in more than 300,000 deaths worldwide in 2020. The expression of the human papillomavirus (HPV) oncogenes E6 and E7 is significantly involved in the initiation and progression of cervical neoplasia. Additionally, the composition of the vaginal microbiome was also closely associated with the ability of HPV to induce cervical cancer. However, the relationship between the expression of HPV E6/E7 oncogene and the composition of the vaginal microbiome has not been clearly explored. In our present study, to investigate the relationship between the HPV E6/E7 oncogene and vaginal microbiome, cervical swabs from 115 patients were collected, and their vaginal microbiomes were analyzed by using metagenomics sequencing. Along with the progression of cervical lesions, the diversity of cervical flora increased gradually, and the abundance of *Lactobacillus* decreased. Compared with the HPV group, the prevalence of HPV E6/E7 and oncogene expression level were significantly upregulated in cervical intraepithelial neoplasia (CIN) and cervical cancer (CC) groups. Additionally, a positive correlation between the expression of the HPV oncogene and the genera *Sneathia*, Salmonella, *Leptotrichia*, Pseudomonas, and *Roseovarius* in the HPV group was observed. In the CIN group, the enrichment of the genera *Sneathia* and *Megasphaera* was weakly linked with HPV oncogene overexpression. In the CC group, a strong association between the overabundance of the genera *Peptostreptococcus* and *Enterococcus* and the high expression of HPV oncogene was also found. The increased diversity of the vaginal microbiota and the decreased *Lactobacillus* abundance were significantly associated with the severity of cervical disease, and the expression of the HPV oncogene could also be regulated by certain pathogens in different stages of cervical lesions.

**IMPORTANCE** The main findings of this study were that we clarified the associations of the different bacterial species with the expression of human papillomavirus (HPV) oncogenes at different stages of cervical cancer. Along with the severity of cervical lesions, the abundance of the genus and species of *Lactobacillus* obviously declined, while the aerobic and anaerobic bacteria, as well as the prevalence and expression of HPV E6/E7 oncogene, were increased dramatically.

## INTRODUCTION

Based on the statistics from the Global Cancer Statistics 2020 report, cervical cancer (CC) was considered the fourth female malignant tumor in both morbidity and mortality (1). The results of the etiology have revealed that theinfection with high-risk human papillomavirus (hrHPV) is the leading cause of cervical lesions and cervical can cer (2). However, due to the elimination of human papillomavirus (HPV) by natural immunity within 2 years after exposure, most HPV infections are considered like a common “cold.” Only less than 1% of persistent patients with hrHPV will eventually develop cervical cancer. Thus, the presence of hrHPV viral DNA is not the only factor necessary to induce cervical cells to become cancerous. Recent evidence demonstrated that HPV integration, rather than transient HPV infection, is a key factor in the initiation and progression of cervical neoplasia (3). Only when a large amount of the HPV oncoproteins E6 and E7 are expressed, the epithelial cell undergo atypical hyperplasia (4). Genetic predisposition, exposure to other sexually transmitted diseases, and the inflammatory microenvironment of the cervical region have been reported to be involved in the integration of HPV genome and the progression of cervical cancer (5).

In recent years, a considerable amount of literature suggested that the composition of the vaginal microbiota is tightly connected to the HPV infection and thus resulting in cervical cancer (6, 7). The predominant presence of the genus *Lactobacillus* in the vaginal microbiome is highly correlated with the elimination of HPV infection and significantly inhibited the development of cervical cancer. Furthermore, the vaginal microbiome dominated by a variety of *Lactobacillus* is considered healthy and positively correlated with microecological functions. Studies showed that the metabolites produced by *Lactobacillus* may participate in local cellular innate immunity, thus inhibiting the infection and replication of the HPV virus (8, 9). However, the depletion of *Lactobacillus* from the vaginal microenvironment and the replacement of them by anaerobes, such as Gardnerella vaginalis, *Prevotella*, and Atopobium vaginalis, were closely associated with the persistent infections of hrHPV (10, 11), cervical hyperplasia (12), increased cervical inflammation, and/or increased infection rates of bacterial vaginitis and HIV (13). A longitudinal cohort study confirmed that the flora of the genital tract is significantly connected to the regression of cervical intraepithelial neonatal (CIN). Women with precancerous lesions dominated by lactic acid bacteria are more likely to undergo regression changes within 1 year, but *Lactobacillus* depletion and specific anaerobic overabundance, including *Macrococcus*, *Prevotella*, and *Gardnerella*, were closely linked with the slower elimination of cervical lesions (14).

However, the relationship between the expression of HPV E6/E7 oncogene and vaginal flora has not been well explored. Previous research was based on the methods of high-throughput 16S sequencing and explored the correlation between the status of HPV infections and the composition of vaginal flora. In this study, the association between the expression of HPV oncogene and the composition of bacteria was determined, and the changes in the composition of cervical flora and the expression of hrHPV oncogene, which significantly correlated with the development of precancerous lesions and cervical cancer caused by hrHPV infection, were also investigated.

## RESULTS

### Taxonomic characterization of cervical microbiota.

The samples collected from a total of 115 patients were sequenced, and then these patients were classified into following three categories: hrHPV positive without cervical lesion category (*n* = 34), precancerous lesions with hrHPV category (*n* = 40), and invasive cervical cancer category (*n* = 41). In addition, all patients were divided into HPV oncogene-negative (*n* = 29) and -positive (*n* = 86) groups (see Table S6 in the supplemental material) for further analysis, ignoring the severity of cervical lesions. After quality control, 8.34 billion 150-bp paired-end clean reads were obtained, and the average reads for each sample were 7.25 ± 0.91 billion. After removal of human DNA contaminants, 11.3 million microbiome reads were generated, with a median of 117.87 (47.08 to 598.24) thousand reads per sample (see Table S1 in the supplemental material).

**FIG 1:**
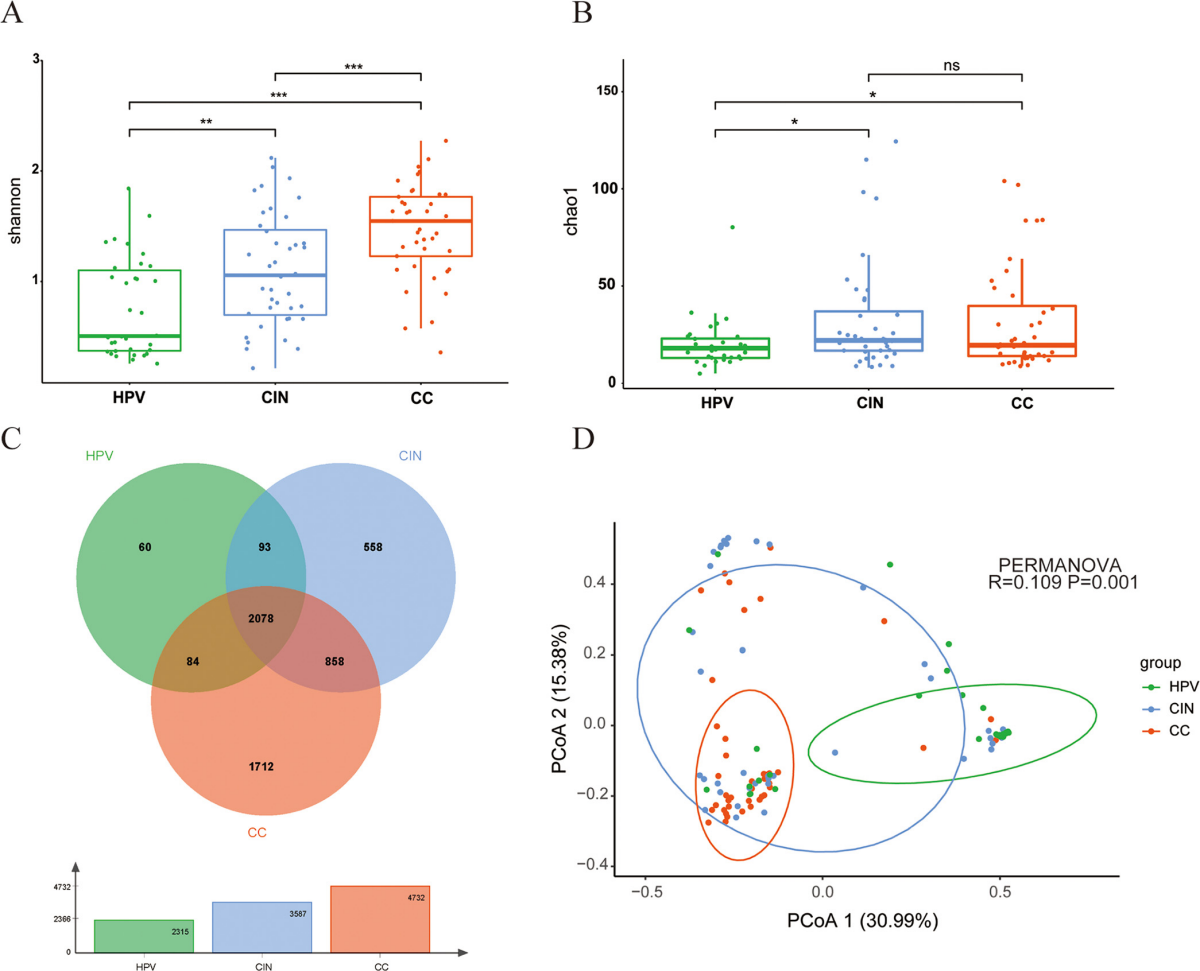
The alpha and beta diversity between the HPV, CIN, and CC groups. (A) Shannon index among three groups. (B) Chao1 index among the three groups. (C) Total species among the three groups. (D) Principal-coordinate analysis among the three groups (PERMANOVA analysis). *, *P* < 0.05; **, *P* < 0.01; and ***, *P* < 0.001.

Then, the alpha diversity in each group was evaluated using Shannon and Chao1 indices according to the species level. As shown in Fig. 1A, we observed significantly high values of the mean Shannon indexes in the patients of the CIN and CC groups compared with those of the HPV group (*P* < 0.001). Moreover, we also observed significantly low Chao1 indexes in the patients of the HPV infection group compared with those of the CIN and CC groups (*P* < 0.001), whereas no significant difference was found between the patients of the CIN and CC group (Fig. 1B). Additionally, along with the progression of the cervical lesions, the total number of microbial species in each group also significantly increased (2,315 species in the HPV group, 3,587 species in the CIN group, and 4,732 species in the CC group) (Fig. 1C). These results supported the finding that the progression of cervical lesions was positively correlated with the diversity of cervical flora. The results from the principal coordinate-analysis (PCoA) plot using the Bray-Curtis statistical algorithm showed that the difference of the beta diversity among the three groups is statistically significant (permutational multivariate analysis of variance [PERMANOVA], *R* = 0.109, *P* = 0.001) (Fig. 1D). After all samples were divided into negative and positive HPV oncogene groups, no significant differences of the Shannon index and PCoA results between these two groups were observed, while the patients of the HPV oncogene-positive group showed a significantly high Chao1 index compared with those of the HPV-negative group (see Fig. S2 in the supplemental material).

**FIG 2:**
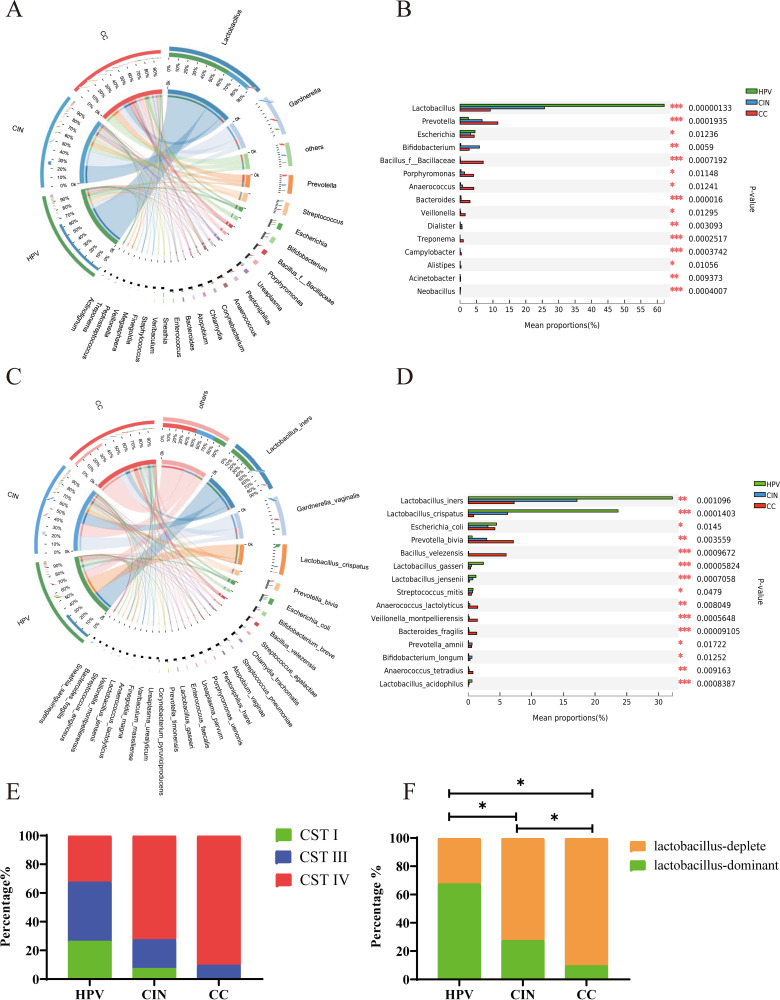
The different composition of the genus and species of the three groups. (A) Circos diagram of the microbial composition at the genus level in the three groups. (B) The significantly different genera in the three groups (left and right semicircles represent different samples and different genus). (C) Circos diagram of microbial composition at the species level in the three groups (left and right semicircles represent different samples and different species). (D) Significantly different species in the three groups. (E) The composition of CST type in the three groups. (F) *Lactobacillus* composition in the three groups. *, *P* < 0.05; **, *P* < 0.01; and ***, *P* < 0.001.

Compared with the patients of the CIN and CC groups, the patients of the HPV group exhibited a significantly high average enrichment of the *Lactobacillus* genus (65.96% in the HPV group, 27.81% in the CIN group, and 9.19% in the CC group), while the average enrichments of the *Gardnerella* genus (7.81%, 24.25%, and 11.24%, respectively), *Prevotella* (2.50%, 6.92%, and 11.69%, respectively), and many other pathogenic bacteria were increased dramatically along with the progression of cervical lesions (Fig. 2A and B). Compared with the patients of the HPV group, the patients of the CIN and CC groups showed a significantly decreased mean abundance of Lactobacillus iners (33.57%, 18.59%, and 7.47%, respectively) and Lactobacillus crispatus (25.73%, 6.99%, and 0.82%, respectively), but the mean abundance of Gardnerella vaginalis (7.72%, 23.85%, and 11.11%, respectively), Prevotella bivia (0.54%, 3.09%, and 6.86%, respectively), and various aerobic and anaerobic bacteria was increased dramatically with the severity of the precancerous lesions (Fig. 2C and D). Vaginal community state types (CSTs) were highly associated with vaginal health, and the dominations of CST I, II, and III by Lactobacillus crispatus, Lactobacillus gasseri, and Lactobacillus iners, respectively, were positively associated with the normal vaginal microbiota. However, CST IV was abundant in anaerobic bacteria, which was associated with bacterial vaginitis, trichomonas vaginitis, CIN, and CC (9, 15). In the HPV group, CST I and CST III accounted for 26.47% and 41.18%, respectively. However, compared with those in the HPV group, the CIN and CC groups showed significantly decreased vaginal CST I (7.5% and 0%, respectively) and vaginal CST III (20.00% and 9.76%, respectively). Moreover, CST IV increased dramatically in the CIN and CC groups (72.50% and 90.24%, respectively) compared with that in the HPV group (32.35%) (Fig. 2E; see Table S2 in the supplemental material). Similarly, along with the progression of cervical lesions, significant reductions of the vaginal microbiome dominated by *Lactobacillus* were also observed (67.65%, 27.50%, and 9.76%, respectively; *P* < 0.05) (Fig. 2F). Significant differences and mean abundances of genus and species in those three groups were presented in Tables S3 and S4, respectively, in the supplemental material. Furthermore, the species with a significant difference between the negative and positive HPV groups mainly included Chlamydia trachomatis, Veillonella montpellierensis, and Enterobacter hormaechei. Contrary to our expectations, no significant differences in the compositional abundance of *Lactobacillus* between these two groups were found (see Fig. S3 in the supplemental material).

### HPV oncogene and blood indicators in different groups.

**FIG 3:**
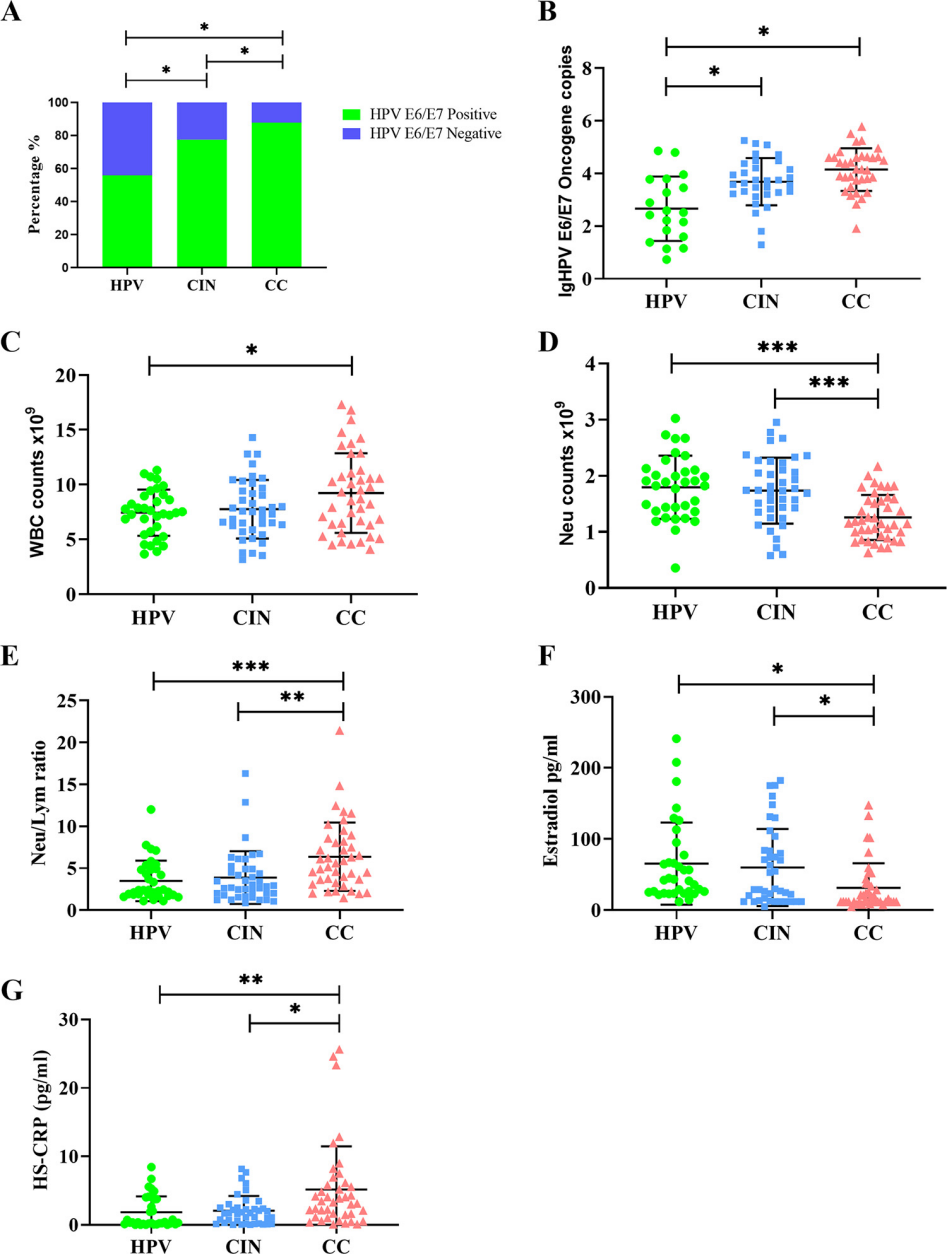
HPV oncogene and blood indicators in different groups. (A) The prevalence of HR-HPV. (B) The expression of the HPV E6/E7 oncogene. (C) The total WBC counts. (D) The total neutrophil counts. (E) The ratio of neutrophils/lymphocytes. (F) The concentration of serum estradiol in the indicated groups of patients. (G) The level of the hypersensitive C-reactive protein in the patients from indicated groups. *, *P* < 0.05; **, *P* < 0.01; and ***, *P* < 0.001.

As shown in Table 1, no significant differences of the age of the patients, the body mass index (BMI), the status of menopause, the liver biochemical indexes, the kidney function, and the serum lipids were observed among the three groups. However, compared with the HPV group, the CIN and CC groups exhibited a significantly high prevalence of HPV E6/E7 oncogene expression (55.88%, 82.05%, and 88.89, respectively; *P* = 0.003) (Fig. 3A). Meanwhile, we also observed an obviously high expression of the HPV E6/E7 oncogene in the CIN and CC groups compared with those in the HPV group (17.84 [0.00 to 1,670.84], 2,871.35 [62.13 to 14,097.28], and 14,425.54 [2,337.19 to 43,039.93], respectively; *P* < 0.001) (Fig. 3B). Additionally, compared with the patients of the HPV group, the total counts of white blood cell (WBC), neutrophil, and hypersensitive C-reactive protein (HS-CRP) levels were significantly elevated in the patients of the CIN and CC group, while the lymphocyte and neutrophil/lymphocyte ratio decreased significantly in samples from patients with cervix lesions (Fig. 3C, D, E and G). Interestingly, compared with the patients with a clinical progression of cancer (CIN or CC), the patients of the HPV group exhibited significantly elevated serum estradiol levels (Fig. 3F). However, no significant differences of the serum inflammatory markers, such as WBC and leukocyte counts and HS-CRP, were found between the negative and positive HPV groups (see Table S6 in the supplemental material).

**TABLE 1:** Clinical baseline characteristics of patients with different cervical cancer progression^*a*^

Characteristic	Data for:			*P* value	*P* value by comparison		
	HPV group (*n* = 34)	CIN group (*n* = 40)	CC group (*n* = 41)		HPV vs CIN	HPV vs CC	CIN vs CC
Age (yrs)	49.74 ± 11.49	50.00 ± 9.95	54.20 ± 7.79	0.099	NS	NS	NS
BMI (kg/m^2^)	22.65 ± 3.20	22.38 ± 3.26	22.72 ± 3.14	0.885	NS	NS	NS
Postmenopause (%)	17 (50.0)	21 (52.5)	28 (68.3)	0.208	NS	NS	NS
Smoker or passive smoker (%)	4 (11.8)	4 (10.0)	6 (14.6)	0.813	NS	NS	NS
Alcohol drink (%)	2 (8.8)	2 (5.0)	5 (12.2)	0.517	NS	NS	NS
RBC (10^12^/L)	4.10 ± 0.42	3.92 ± 0.42	3.85 ± 0.52	0.054	NS	NS	NS
RDW-SD (fL)	43.16 ± 4.28	41.96 ± 3.38	43.61 ± 3.82	0.141	NS	NS	NS
HB (g/L)	118.24 ± 18.65	115.68 ± 16.16	110.49 ± 16.13	0.13	NS	NS	NS
PLT (10^9^/L)	234.32 ± 54.85	210.43 ± 70.33	227.71 ± 92.66	0.365	NS	NS	NS
AST (U/L)	22.12 ± 12.03	19.40 ± 9.14	22.59 ± 9.43	0.54	NS	NS	NS
ALT (U/L)	21.32 ± 14.85	18.28 ± 8.39	19.73 ± 11.67	0.326	NS	NS	NS
GGT (U/L)	14.00 (10.00–22.25)	14.00 (11.00–19.50)	19.00 (11.50–42.50)	0.135	NS	NS	NS
TBIL (μmol/L)	11.09 ± 4.04	12.57 ± 3.85	10.51 ± 3.75	0.053	NS	NS	NS
DBIL (μmol/L)	3.26 ± 1.18	3.68 ± 1.30	3.10 ± 1.19	0.097	NS	NS	NS
TP (g/L)	66.65 ± 5.19	64.96 ± 6.79	65.48 ± 6.54	0.504	NS	NS	NS
ALB (g/L)	41.65 ± 3.98	41.10 ± 4.48	40.13 ± 4.64	0.315	NS	NS	NS
UREA (mmol/L)	4.59 ± 1.02	4.76 ± 1.05	4.62 ± 1.19	0.743	NS	NS	NS
CREA (μmol/L)	54.09 ± 9.69	53.03 ± 11.96	53.85 ± 12.82	0.916	NS	NS	NS
UA (μmol/L)	277.69 ± 82.45	301.63 ± 86.00	292.83 ± 104.23	0.534	NS	NS	NS
C1q (mg/L)	185.85 ± 33.45	180.66 ± 34.52	199.44 ± 43.54	0.074	NS	NS	NS
Glu (mmol/L)	4.84 ± 0.63	4.71 ± 0.68	5.05 ± 1.00	0.149	NS	NS	NS
TC (mmol/L)	4.05 (3.39–4.28)	4.28 (3.79–5.03)	4.18 (3.76–4.75)	0.658	NS	NS	NS
TG (mmol/L)	1.09 (0.83–1.62)	1.15 (0.98–1.56)	1.30 (1.05–2.20)	0.136	NS	NS	NS
HDL-c (mmol/L)	1.28 ± 0.34	1.26 ± 0.24	1.18 ± 0.31	0.277	NS	NS	NS
LDL-c (mmol/L)	2.66 ± 0.82	2.68 ± 0.81	2.38 ± 0.76	0.175	NS	NS	NS
sdLDL (mmol/L)	0.70 ± 0.36	0.77 ± 0.33	0.73 ± 0.36	0.695	NS	NS	NS
HPVE6/E7 oncogene copies	56,973.19 ± 58.82	101,036.48 ± 72.83	289,409 ± 168.78	**<0.001**	**<0.001**	**<0.001**	**<0.001**
Shannon index^*b*^	0.76 ± 0.08	1.10 ± 0.08	1.46 ± 0.07	**<0.001**	**<0.01**	**<0.001**	**<0.001**
Chao1 index^*b*^	19.84 ± 2.18	32.88 ± 4.55	34.03 ± 4.57	**<0.001**	**<0.05**	**<0.05**	NS
WBC (10^9^/L)	7.46 ± 0.36	7.76 ± 0.42	9.22 ± 0.57	**<0.001**	NS	**<0.05**	**<0.05**
Neu (10^9^/L)	5.23 ± 0.39	5.36 ± 0.39	7.21 ± 0.57	**<0.001**	NS	**<0.001**	**<0.001**
Lym (10^9^/L)	1.80 ± 0.10	1.74 ± 0.93	1.26 ± 0.06	**<0.001**	NS	**<0.001**	**<0.01**
Neu/Lym ratio	3.50 ± 0.42	3.89 ± 0.50	6.37 ± 0.64	**<0.001**	NS	**<0.001**	**<0.01**
HS-CRP (mg/dL)	1.83 ± 0.40	2.06 ± 0.34	5.64 ± 1.28	**<0.001**	NS	**<0.01**	**<0.05**

a TC, total cholesterol; TG, triglycerides; HDL-c, high-density lipoprotein cholesterol; LDL-c, low-density lipoprotein cholesterol; WBC, white blood cell; Neu, neutrophil; Lym, lymphocyte; HS-CRP, hypersensitive C-reactive protein. Boldface text indicates significance; NS, no significance.

b Shannon index and Chao1 index, alpha diversity of the cervical microbiome.

### Association between cervical microbiota, HPV oncogene expression, and blood indices.

Next, we conducted the Spearman correlation analysis on these three groups to explore the correlation between the vaginal microbiome and HPV oncogene expression. The results showed that the HPV oncogene expression was strongly correlated with the presence of the genus *Sneathia* (*R* = 0.74, *P* < 0.001), Salmonella (*R* = 0.57, *P* < 0.001), *Leptotrichia* (*R* = 0.64, *P* < 0.001), Pseudomonas (*R* = 0.56, *P* < 0.001), and *Roseovarius* (*R* = 0.63, *P* < 0.001), while a weakly positive correlation of the HPV oncogene expression with the genus *Ureaplasma* (*R* = 0.35, *P* = 0.041) and Klebsiella (*R* = 0.38, *P* = 0.028) was also observed in the HPV group. Additionally, a strong positive association of Sneathia amnii (*R* = 0.74, *P* < 0.001) and Salmonella enterica (*R* = 0.57, *P* < 0.001) with the HPV oncogene expression were found at the species level, while we also observed a weakly positive association of Ureaplasma parvum (*R* = 0.41, *P* = 0.017) and Klebsiella pneumoniae (*R* = 0.38, *P* = 0.028) with the presence of HPV oncogenes. In the CIN group, the high abundance of the genus *Sneathia* (*R* = 0.40, *P* = 0.011) and *Megasphaera* (*R* = 0.43, *P* = 0.006) were weakly associated with the HPV oncogene expression. At the species level, we observed strong associations of Sneathia sanguinegens (*R* = 0.42, *P* = 0.007), *Megasphaera* sp. *UPII_135-E* (*R* = 0.35, *P* = 0.027), *Megasphaera genomosp. type_1* (*R* = 0.36, *P* = 0.022), and *Megasphaera* sp. UPII_199-6 (*R* = 0.36, *P* = 0.024) with the HPV oncogene expression. Additionally, we also observed the strong correlations of the overabundance of genera *Peptostreptococcus* (*R* = 0.71, *P* < 0.001) and *Enterococcus* (*R* = 0.80, *P* < 0.001) with the high expression of the HPV oncogene, as well as the weak association between the *Parabacteroides* (*R* = 0.34, *P* = 0.031) and the overexpression of the HPV oncogene, Meanwhile, the associations of Peptostreptococcus anaerobius (*R* = 0.71, *P* < 0.001), Enterococcus faecalis (*R* = 0.79, *P* < 0.001), Bacteroides thetaiotaomicron (*R* = 0.83, *P* < 0.001), and Porphyromonas uenonis (*R* = 0.40, *P* = 0.009) with the HPV oncogene expression were also discovered at the species level.

**FIG 4:**
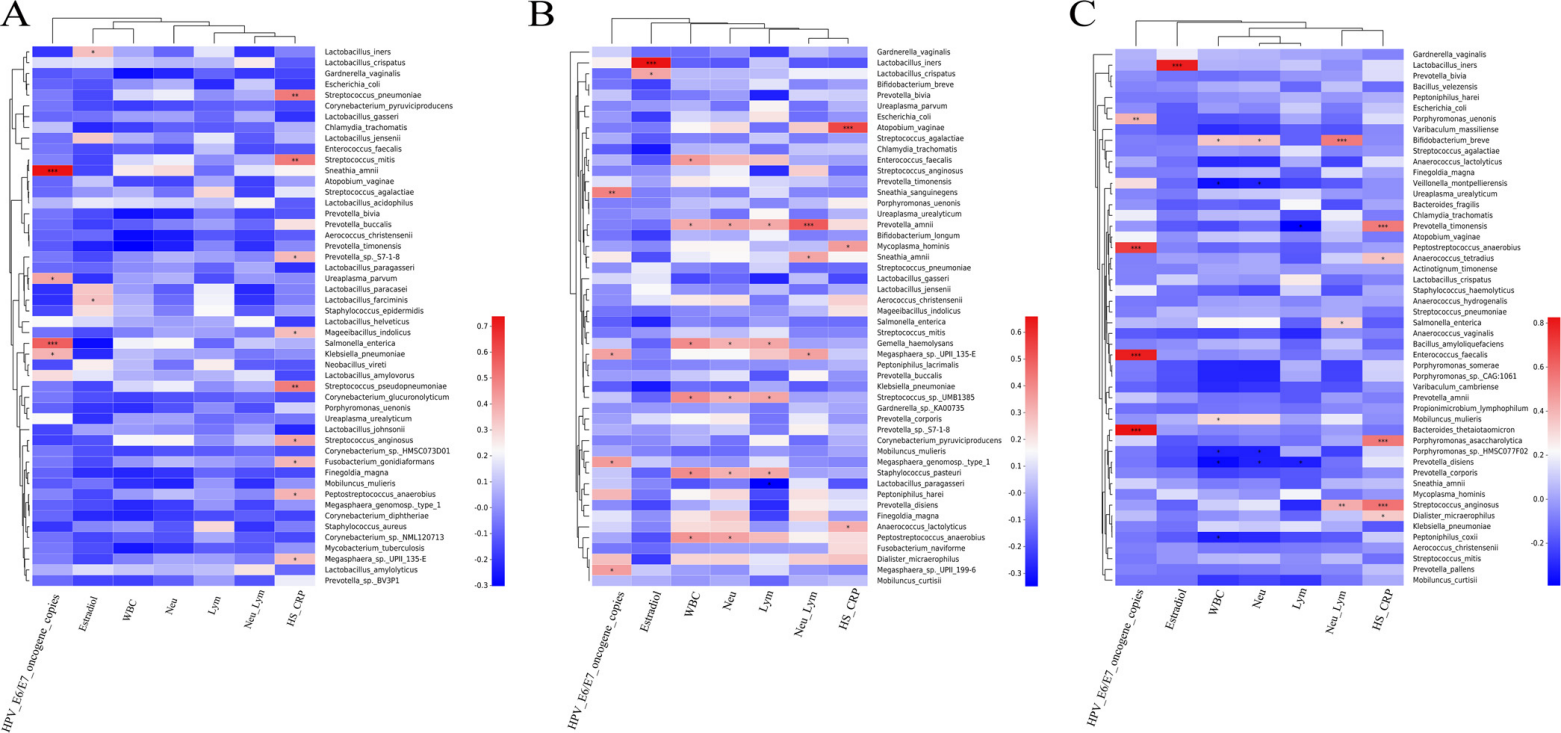
Correlations between microbiota species, HPV oncogene expression, and blood indices. (A) HPV group. (B) CIN group. (C) Cervical cancer group. Spearman rank correlation coefficient is indicated using a color gradient, as follows: red indicates a positive correlation and blue indicates a negative correlation. *, *P* < 0.05; **, *P* < 0.01; and ***, *P* < 0.001.

We further explored the correlations between the vaginal microbiome and blood indexes. The results showed that the serum estradiol level was positively associated with the cervical enrichment of the *Lactobacillus* genus among the three groups (all *R* > 0.3, *P* < 0.05), but weakly associated with the *Neobacillus* (*R* = 0.68, *P* < 0.001) in the CIN group and *Mycoplasma* (*R* = 0.49, *P* = 0.001) in the CC group (see Fig. S1 in the supplemental material). In line with the results of the genus level, significantly positive associations of the serum blood estrogen with the high abundance species of Lactobacillus iners (all *R* > 0.3, *P* < 0.05) among the three groups, Lactobacillus farciminis (*R* = 0.34, *P* = 0.046) in the HPV group, and Lactobacillus crispatus (*R* = 0.34, *P* = 0.032) in the CIN group were also found. Additionally, in the patients of the HPV group, significantly positive correlations of the WBC count with the genera *Enterococcus*, *Anaerococcus*, *Gemella*, Staphylococcus, *Peptostreptococcus*, *Bacillus_f__Bacillaceae*, *unclassified_p__Candidatus_Saccharibacteria*, and *Clostridium* were discovered. Furthermore, in the CC group, we simultaneously observed a positive association of the WBC and neutrophil with the genus *Bifidobacterium* and a negative association with the genus *Veillonella*. As shown in Fig. 4, the HS-CRP showed significantly positive correlations with the various aerobic and anaerobic species, such as Streptococcus pneumoniae, Streptococcus mitis, *Prevotella sp. S7-1-8*, Mageeibacillus indolicus, Streptococcus pseudopneumoniae, Streptococcus anginosus, Fusobacterium gonidiaformans, Peptostreptococcus anaerobius, and *Megasphaera sp.UPII_135-E* in the HPV group; Atopobium vaginae, Mycoplasma hominis, and Anaerococcus lactolyticus in the CIN group; and Prevotella timonensis, Anaerococcus tetradius, Porphyromonas asaccharolytica, Streptococcus anginosus, and Dialister micraerophilus in the CC group (Fig. S1). Interestingly, when we conducted a similar correlation analysis in the HPV oncogene-positive group, a positive association of the HPV oncogene expression and Peptostreptococcus anaerobius and Bacteroides thetaiotaomicron was observed again, suggesting the close relation of these bacteria to the progression of cervical cancer. Furthermore, we also found a significantly negative correlation between the abundance of L. crispatus *and L. iners* and the alpha diversity in HPV oncogene-positive and -negative groups (see Fig. S5 in the supplemental material).

### Identification of CIN and CC patients based on cervical microbiota.

**FIG 5:**
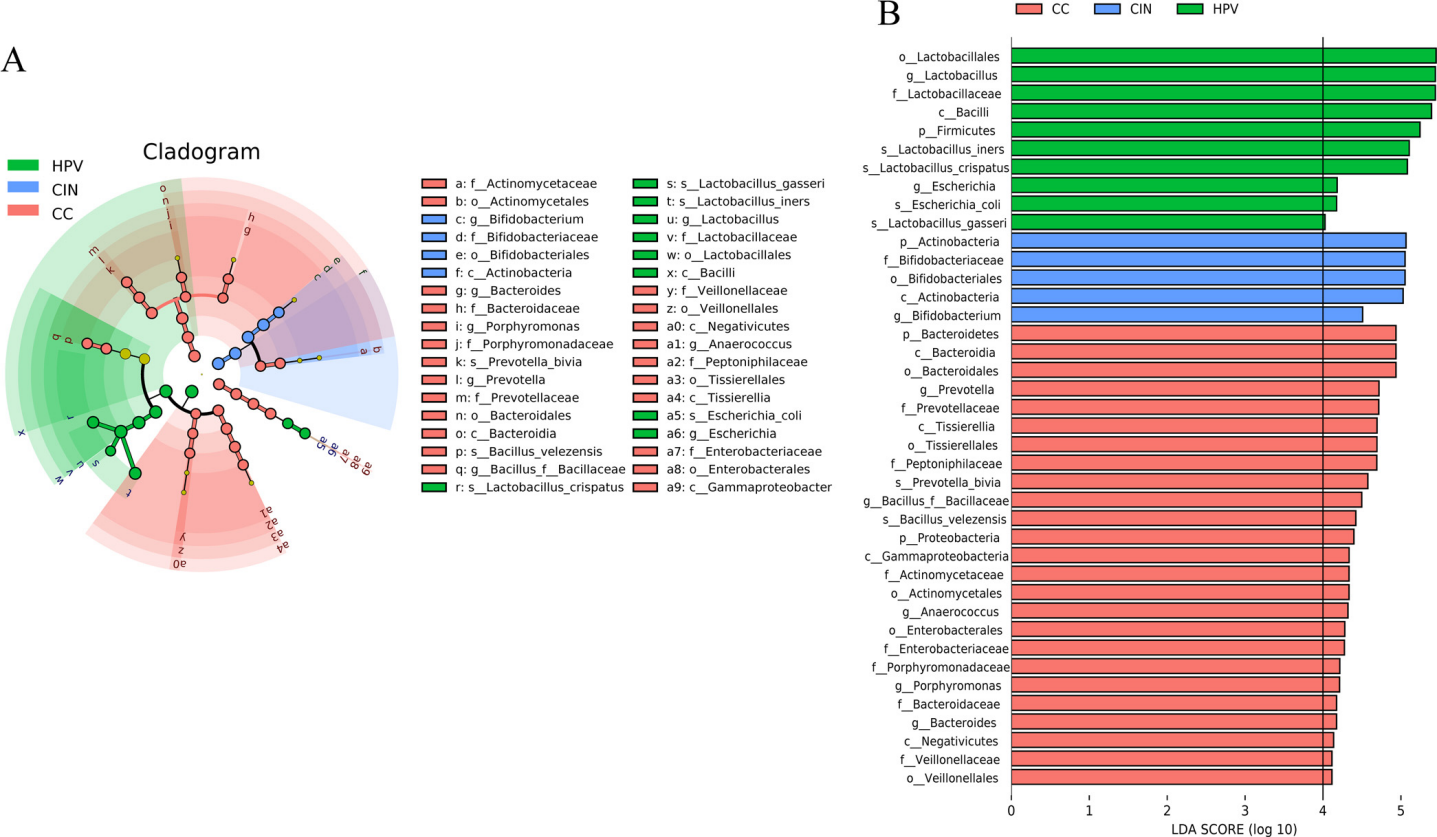
The LDA effect size analysis (LEfSe) results to classify the underlying biomarkers revealed the variety of the cervical microbiome based on the cervical lesion status. (A) Differentially enriched microbial clades and nodes are shown in a cladogram. The circles radiating from the inside out represent the taxonomic level from phylum to species. Each small circle at a different taxonomic level represents the taxonomy at that level, and the diameter of the small circle is proportional to the relative abundance. (B) Microbes of differential abundance were plotted as a histogram with an LDA score > 4. The lengths of the bars represent the effect sizes of species that significantly distinguish HPV, CIN, and CC groups.

**FIG 6:**
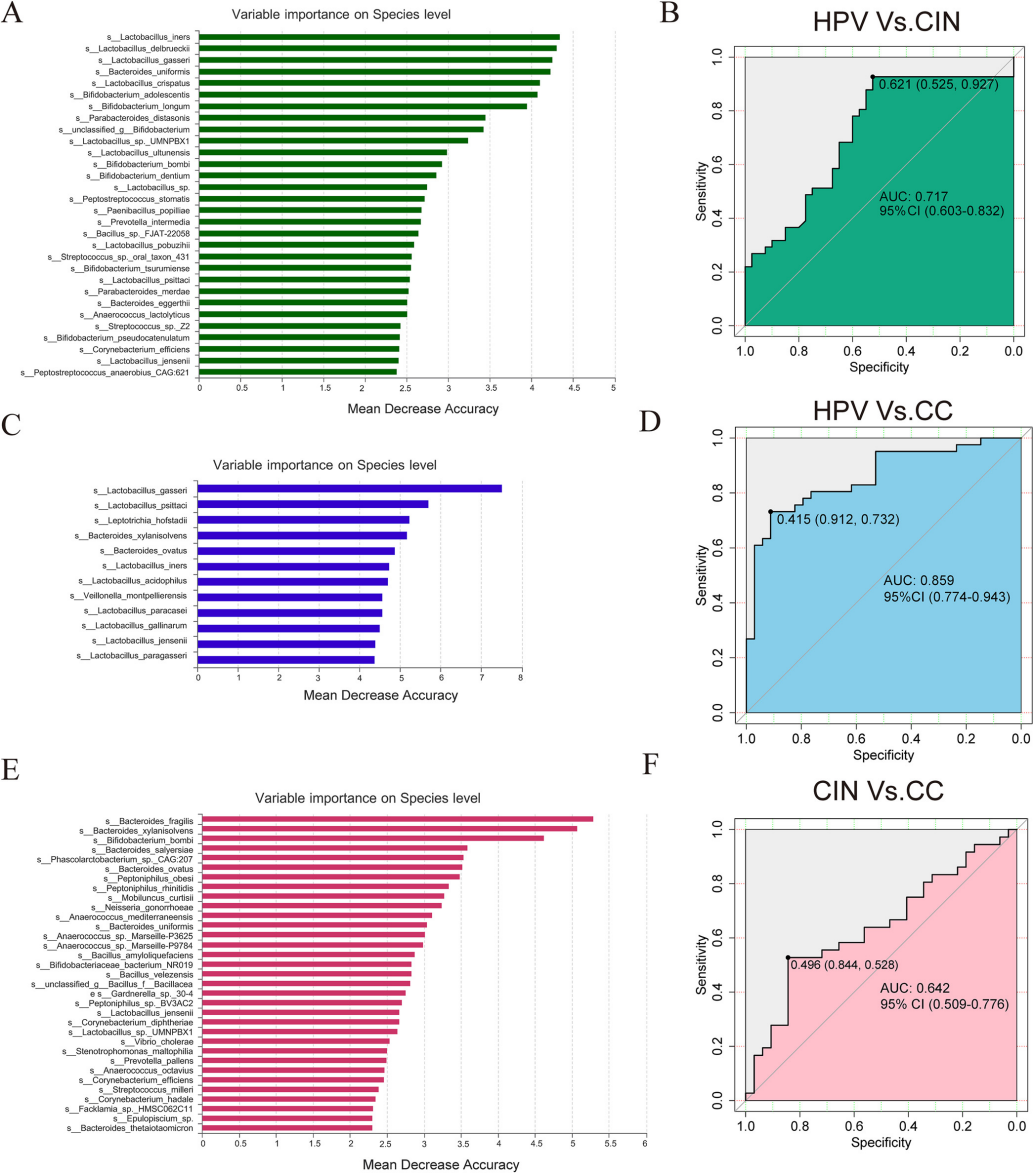
Identification of markers based on microbial species of HPV, CIN, and CC by random forest models. The microbial biomarkers were identified to construct random forest models for discriminating each of two groups. (A) HPV versus CIN (top 30 important species); (C) HPV versus CC (top 12 important species); (E) CIN versus CC (top 33 important species). The AUCs of the optimized models were constructed with microbial biomarkers of each of the two groups. (B) HPV versus CIN; (D) HPV versus CC; (F) CIN versus CC.

Linear discriminant analysis (LDA) effect size (LEfSe) modeling was conducted to distinguish differences in bacterial abundance that might be linked to the progression of cervical lesions (Fig. 5; Table S5). We have restricted the LDA score of ≥4 as showing effective biomarkers. We found that Lactobacillus iners, Lactobacillus crispatus, Escherichia coli, and Lactobacillus gasseri were considered potential biomarkers for identifying HPV infection to any cervical lesions. Additionally, the *Bifidobacterium* genus was treated as an excellent microbiota biomarker for precancerous patients. Also, *Prevotella*, *Porphyromonas*, and *Bacteroides* genera were considered great microbial markers of the cervical cancer group. After performing 10-fold cross-validation on random forest models between every two groups as a previous study described (16), we found several most important species that could be treated as potential biomarkers for distinguishing the HPV, CIN, and CC patients (Fig. 6), similar to the LEfSe results. The receiver operating characteristic (ROC) curve of optimized microbial biomarkers could effectively distinguish the HPV group from CIN patients (area under the concentration-time curve [AUC], 0.717; 95% confidence interval [CI], 0.603 to 0.832) and CC patients (AUC, 0.859; 95% CI, 0.7740 to 0.943). However, it was difficult to classify the CIN and CC patients (AUC, 0.642; 95% CI, 0.509 to 0.776). Additionally, we also found several microbial biomarkers to predict the HPV oncogene-positive patients, such as species of Chlamydia trachomatis, Veillonella montpellierensis, Bifidobacterium longum, and Bifidobacterium bifidum (see Fig. S4 in the supplemental material).

## DISCUSSION

A complete understanding of the relationships between microorganisms and the progression of cancer has not been clearly explored; however, a variety of microbiota members participate in the development of cancer, specifically cervical cancer (9). The infection of HPV as the major pathogenesis of cervical cancer has been well established. However, the association between the vaginal microbiome and HPV oncogene expression has not been determined. In the present study, using the metagenomic sequencing and analysis, we explored the change of the vaginal microbiome throughout cervical lesion progression and determined the association between the progression of cervical lesions with HPV oncogene expression.

Consistent with the previous studies, we observed that the depletion of the genus *Lactobacillus* and *Lactobacillus* spp. was significantly associated with the progression of cervical cancer (12, 17). Previous mechanistic studies have shown that, through inhibiting the expression of the HPV E6/E7 oncogene, suppressing the proliferation and metastasis of the cancer cells, and promoting the apoptosis, *Lactobacillus* or *Lactobacillus* supernatant could significantly inhibit the progression of cervical cancer *in vitro* and *in vivo* (18–20). Furthermore, through producing antimicrobial agents, competitively inhibiting the adhesion of pathogenic bacteria and viruses, and maintaining a low pH and local anti-inflammatory microenvironment (21), the dominant *Lactobacillus* community state types (CST I, CST II, and CST III) could effectively maintain a healthy vaginal environment, which could exert a vital role in the elimination of HPV infection (22, 23). Furthermore, a recent study using transgenic mice showed that HPV oncoproteins can greatly inhibit the expression of host defense peptides, which were the source of essential amino acids for the survival of vaginal commensal *Lactobacillus*, downregulating these innate immune molecules that ultimately lead to dysbiosis of the vaginal microbiota (24). This novel viral immune evasion strategy mechanistically explained why vaginal lactobacilli tended to be depleted when more HPV oncogenes were expressed.

In line with previous reports, the diversity of species in the vaginal microenvironment increased with the progression of cervical lesions, and the *Gardnerella*, *Prevotella*, Streptococcus, and many other anaerobe genera increased gradually with the development of cervical cancer (15, 25). Likewise, CST IV (the cervical type dominated by these pathogenic bacteria) was also more prone to cervical lesions (26). It is worth noting that the function of different types of *Lactobacilli* in the vaginal microbiota remains controversial. The vaginal microbiota dominated by L. crispatus and L. gasseri was dramatically involved in the maintenance of a healthy vaginal microenvironment. However, the vaginal microbiota dominated by *L. iners* was often considered a nonprotective effect but closely associated with the increased vaginal inflammation and the increased likelihood of conversion to bacterial vaginosis (27).

Our results have shown that the expression of the HPV oncogene is positively associated with the various species of bacteria, especially with the genera *Sneathia* and *Peptostreptococcus.* Sneathia sanguinegens is a close species of Fusobacterium nucleatum, which significantly participated in the initiation and progression of colorectal cancer by various underlying molecular mechanisms, such as immune regulation, adhesion proteins production, and microRNAs (28, 29). Additionally, through promoting the metastasis of cancer cells, the high intertumoral abundance of Fusobacterium nucleatum was also associated with the poor prognosis of cervical cancer (30). Furthermore, Peptostreptococcus anaerobius, a known cancer-driver pathogen in colorectal cancer, is significantly involved in the regulation of immune responses and the promotion of colorectal carcinogenesis (31). However, the underlying molecular mechanisms about how these bacterial species participate in the development of cervical cancer and the expression of HPV oncogene need further investigation.

In this work, we observed that the presence of *Lactobacillus* and *Lactobacillus* sp. was positively correlated with the serum estradiol level in the three groups. Previous research demonstrated that serum estradiol could promote the production of glycogen in cervical epithelial cells, which provides the nutrition for *Lactobacillus* growth and supports multiple aspects of vaginal health (32). Furthermore, the intravaginal estrogen treatment could significantly repress and clear the precancerous lesions in the patients with vaginal intraepithelial neoplasia (33). In addition, estradiol administered by vaginal supplement is considered an effective treatment for recurrent vaginitis (34). The cervical estradiol supplements might provide a potential therapeutic strategy for HPV-positive cervical cancer (35), but more clinical trials to confirm its therapeutic effect are needed.

According to a previous study, the total count of WBCs and the number of neutrophils significantly increased in the patients of the CIN and CC groups, while the ratio of neutrophil/lymphocyte is dramatically decreased in the cervical lesion groups (36). Similarly, the patients of the CIN and CC groups exhibited a significantly high HS-CRP level compared with the patients of the HPV group, suggesting severe inflammation in the patients with cervical lesions. Furthermore, along with the severity of cervical lesions, dramatically elevated interferon gamma (IFN-γ) production in the cervical mucus was discovered by a previous study (37). Because of the high abundance of Streptococcus, *Prevotella*, *Mageeibacillus*, *Fusobacterium*, *Peptostreptococcus*, *Megasphaera*, and *Atopobium*, the severe systemic and local inflammations were closely associated with HPV expression of the oncogene and vaginal microbiome dysbiosis (38).

The HPV DNA test combined with the thin-prep cytology test (TCT), colposcope, and biopsy were generally employed in the screening and diagnosis of the patients with CIN and CC in the clinic. Recently, noninvasive microbiome models of the intestinal tract for the diagnosis and prediction of many types of cancer, such as colorectal, breast, and liver cancer, have been established (39). Similarly, we hypothesized that the compositions of the vaginal microbiome may be another good biomarker for the prediction of the patients with CIN and CC. Consistent with previous studies, we considered that *Lactobacillus* spp. can be used as a good biomarkers for the prediction of HPV infection, and the various pathogenic anaerobic and aerobic bacteria, such as *Bacteroidetes*, *Prevotella*, and *Prevotellaceae*, can serve as potential biomarkers for the prediction of cervical lesions (11, 40). Interestingly, the species markers for each stage of cervical cancer were different. Also, the species correlated with the expression of viral oncogenes were distinct, suggesting the different bacteria possibly connected with different functions at different cancer stages (41). Thus, compared with the HPV DNA test which was used in previous studies, our data demonstrated that the vaginal microbiome has a significant value for the diagnosis of the precancerous lesions and cervical cancer (42, 43). The HPV oncogene combined with microbiome models for the classification of HPV, CIN, and CC patients need further investigation and evaluation using clinical trials.

### Strengths and limitations.

We conducted this comprehensive study to reveal the potential relationship between cervical lesions, cervical microbiota, and HPV oncogene expression using metagenomics sequencing. The current study also has several shortcomings that limit the conclusions that can be drawn. First, the study size number was small and did not have a validation cohort. A large and multicenter verification is required for confirming the conclusion of the current study. On the other hand, due to the contamination of human host DNA, effective sequencing data of vaginal microorganisms are limited, and more depth-sequencing methods that remove the human host genome are required to validate our results. Furthermore, the genotype of hrHPV was not equivalent in each group, and since, here, we conducted a cross-sectional study, a longitudinal cohort is needed to verify our result. Furthermore, in our present study, several important indicators of vaginal inflammation were not included, and the variables, such as vaginal pH and Nugent score, should be included in future studies. Lastly, our study demonstrated the potential relationship between the HPV oncogene, the cervical microbiota, and the development of cervical cancer, but the causality and underlying molecular mechanisms of these three aspects still need to be further investigated.

### Conclusions.

Along with the severity of cervical lesions, the abundance of the genus *Lactobacillus* and species was reduced and the abundance of anaerobic and aerobic bacteria was increased, while the prevalence of HPV E6/E7 and the expression of oncogenes were elevated. The HPV oncogene overexpression was associated with distinct bacterial species during different stages of cervical cancer. More studies are needed to explore the underlying molecular mechanisms of different pathogens that can inhibit or advance the formation of cervical lesions.

## MATERIALS AND METHODS

### Study design and specimen collection.

All nonduplicated patients were recruited from June 2020 to March 2021 at Renmin Hospital of Wuhan University and had a Han Chinese background. These patients were pathologically diagnosed with precancerous lesions or invasive cervical cancer for the first time. Additionally, patients met the following criteria: (i) not received cervical and vaginal surgery or chemoradiation, (ii) not consumed oral contraceptives and/or antibiotics in 3 months, (iii) not received hormone replacement therapy in 1 year, (iv) absence of vaginal lavage or sexual intercourse within 24 h before sample collection, and (v) must not have been diagnosed with any other gynecological tumors and immunosuppressive diseases. Furthermore, patients with hrHPV infection without cervical lesions were used as the HPV infectious group. All negative cervical lesions of the control populations were confirmed by TCT and colposcopy.

**FIG 7:**
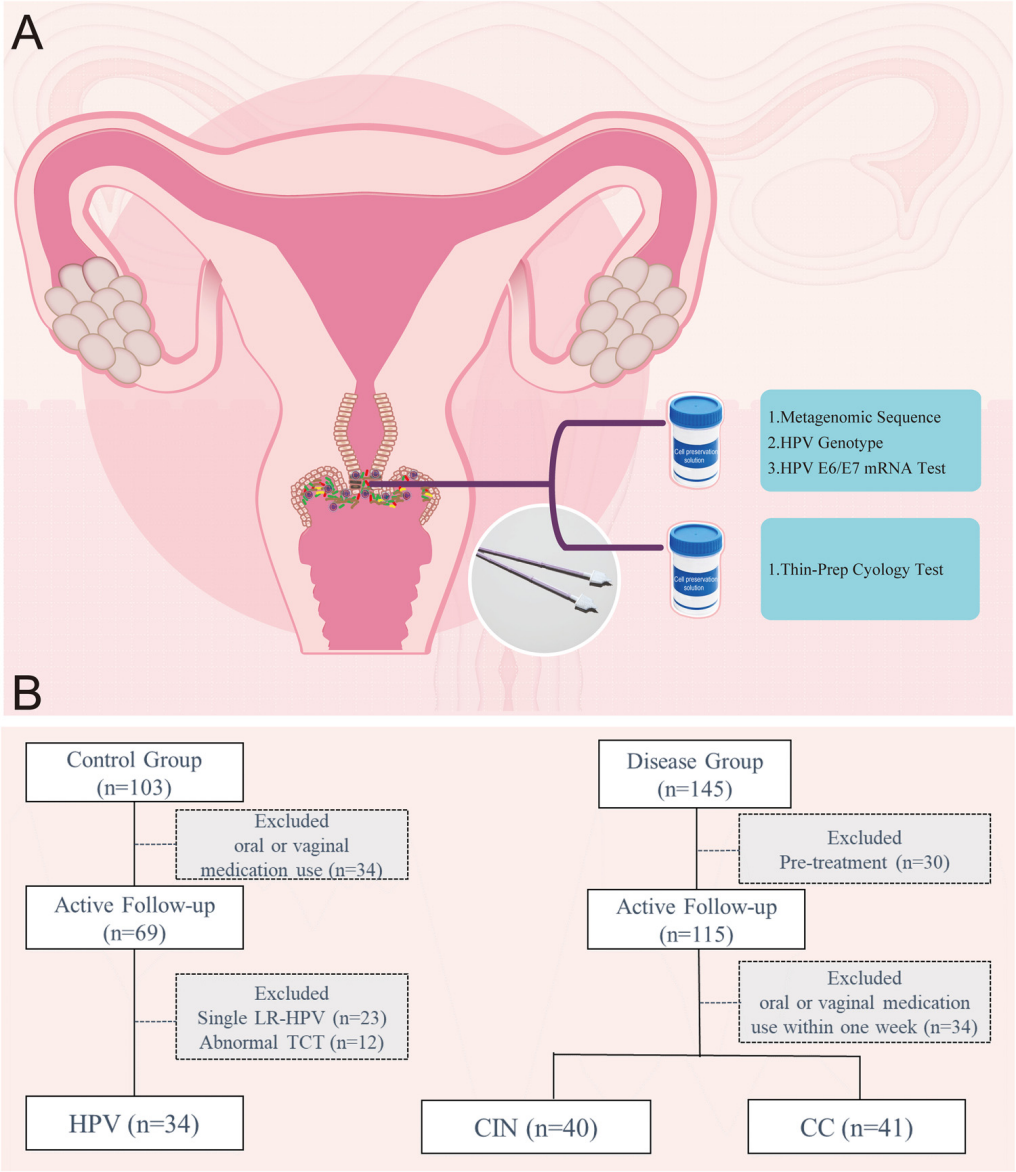
Flow chart of patients and samples collected in this study. (A) Collection of cervical microbiota samples from the cervical transformation zone. (B) Patient screening process for metagenomic sequencing.

Cervical samples were collected according to the standard procedures (Fig. 7). All samples were preserved in the cell preservation fluid and then immediately frozen at −80°C until DNA or RNA extraction. Fasting venous blood was drawn and centrifuged at 3,300 rpm for 6 min and then stored in an ultralow temperature freezer until follow-up detection. The postmenopausal period was defined as age over 55 years or normal amenorrhea of more than 12 months. The blood and cervical brushes were collected from the premenopausal patients between 3 and 7 days after menstruation.

### Nucleic acid extraction, HPV genotype, and E6/E7 oncogene mRNA detection.

The Qiagen DNeasy PowerSoil pro kit (catalog [cat.] number 47014) was used to extract DNA from cytobrush samples with several modifications. Briefly, the cervical brush was vigorously vortexed with cell preservation fluid for 5 min, and then 1 mL of the mixed liquid was pipetted into a new tube and centrifuged for 15 min at 15,000 rpm. Nucleic acids were extracted according to the kit instructions. The RNA was extracted by adopting the magnetic total RNA extraction kit (cat. number NAE-13; Biotron, Guangzhou, China). The optical density of DNA and RNA 260/280 was detected using a Nanodrop 2000c spectrophotometer (Thermo Fisher Scientific, USA).

HPV genotypes were detected according to the manufacturer’s handbook (Tellgenplex HPV27 genotyping test). The hrHPV infection was defined as any infectious HPV genotype of 16, 18, 26, 31, 33, 35, 39, 45, 51, 52, 53, 56, 58, 59, 66, 68, and 82. The expression of the HPV E6/E7 oncogene mRNA was determined using the high-resolution melt curve method (Papilloplex hrHPV genotyping kit; Biotron, Guangzhou, China). The copies of HPV E6/E7 per 1,000 cells were measured using a single reference gene marker, HPRT, and the relative expression of the HPV E6/E7 gene was calculated using the threshold cycle (2^−ΔΔCT^) method. Thin-prep cytology test (TCT) samples were prepared by the AZR-D and ATP-E liquid-based cytology machine (Xiaogan Aohua Medical Technology Co., Ltd.) according to the manufacturer’s instructions.

### Metagenomic shotgun sequence and data analysis processing.

Cervical microbiome species were sequenced using the Illumina HiSeq 2500 platform (Chunlab, Inc., Seoul, South Korea), and paired-end reads were generated with 150 bp. The Fastp software (version 0.20.0) was applied to trim the 3′ end and 5′ end of the barcode sequence and to remove sequences with a length less than 50 bp and an average quality value lower than 20, the unidentified bases after mass shearing, the reserve high-quality pair-end reads, and the single-end sequences. Before the data analysis, sequence reads were compared with the human host DNA sequence using the software BWA (version 0.7.9a), and the host genome-contaminated reads with high similarity were removed. The remaining high-quality reads were assembled using Multiple Megahit (version 1.1.2), and the shortest contigs were over 300 bp. The MetaGene was used to predict open reading frames (100 bp) from previously assembled contigs. The gene sequences predicted by the sample were clustered by CD-HIT software to constitute a nonredundant gene catalog and to obtain the base sequence of the gene of the nonredundant gene catalog (identity, <90%; coverage, <90%). To align the high-quality reads of each sample with the nonredundant gene database (identity, <95%) and to calculate the abundance information of the genes in the matching sample, the SOAPaligner software was applied.

In order to align the nonredundant gene catalog, the DIAMOND software was applied with the nonredundant (NR) database (blastp, E value of ≤1e^−5^), and the species annotation result was obtained using the taxonomic information database matching the NR library. Then the counts of each species were calculated using the total gene number corresponding to the species, and the counts of each species were calculated as the domain, kingdom, phylum, class, order, family, genus, and species at each taxonomic level to count the abundance of species in each sample to construct the corresponding classification abundance profile at the taxonomic level. To avoid interference from the interkingdom species, the genes annotated to nonbacteria, such as fungi, viruses, and parasites, were excluded from the final nonredundant gene set. As described previously (44), the reads per kilobase million (RPKM) method was applied to adopt the relative abundance of genes and the annotated species.

### Clinical data collection and serum index detection.

The clinical baseline data of all enrolled patients, such as menstrual status and history of drinking, smoking, and medication treatments, were collected from medical records. Serum estradiol was detected according to our previous study using liquid chromatography-tandem mass spectrometry (LC-MS/MS; Applied Biosystems, Foster City, CA) (45). Serum biochemical indicators, such as aspartate aminotransferase (AST), UREA, CREA, and hypersensitive C-reactive protein (HS-CRP), were determined using a Siemens ADVIA 2400 automated clinical chemistry analyzer (Erlangen, Germany). Hemocyte counts were determined using the automatic blood analyzer system (SysmexXN-20; Kobe, Japan).

The samples and clinical data collected in the present study were approved by the Medical Ethics Review Committee of Renmin Hospital, Wuhan University, China (ethics record number WDRY2021-K042). All patients signed informed consent. We confirmed that all methods were carried out in accordance with the Declaration of Helsinki and relevant local and national guidelines and regulations.

### Statistical analysis.

The types of vaginal microbiome were classified as described previously (14). The bioinformatic analysis was performed using the Majorbio Online platform and the R package (R version 4.1). The alpha diversity of the Shannon and Chao1 indexes was calculated using the vegan package. The beta diversity was conducted by adopting the principal-coordinate analysis (PCoA; Bray-Curtis algorithm). Random forest models were conducted by using the R package (version 4.6-14), and the ROC curves were plotted by the pROC package (version 1.17.0). The chi-square test or Fisher’s exact test was performed to compare categorical variables, and they were expressed in frequency and percentage. According to whether the data were normally distributed, the one-way analysis of variance (ANOVA) test or Kruskal-Wallis H test was to compare continuous variables in multiple comparisons. The Tukey-Kramer method was applied for the *post hoc* test and a two-way *P* value of <0.05 was considered statistically significant.

### Data availability.

All raw data from the metagenomics sequence have been uploaded to the NCBI Sequence Read Archive database (accession number: PRJNA771720).
