# Supplementary material for: Association of Cervical Dysbacteriosis, HPV Oncogene Expression, and Cervical Lesion Progression
Source: Microbiol Spectr. 2022 Aug 29;10(5):e00151-22. doi: 10.1128/spectrum.00151-22 (PMC9602310; doi:10.1128/spectrum.00151-22)
Supplement: Supplemental file 1 — Fig. S1-S5;Tables S1-S6. Download spectrum.00151-22-s0001.pdf, PDF file, 2.6 MB [file spectrum.00151-22-s0001.pdf]

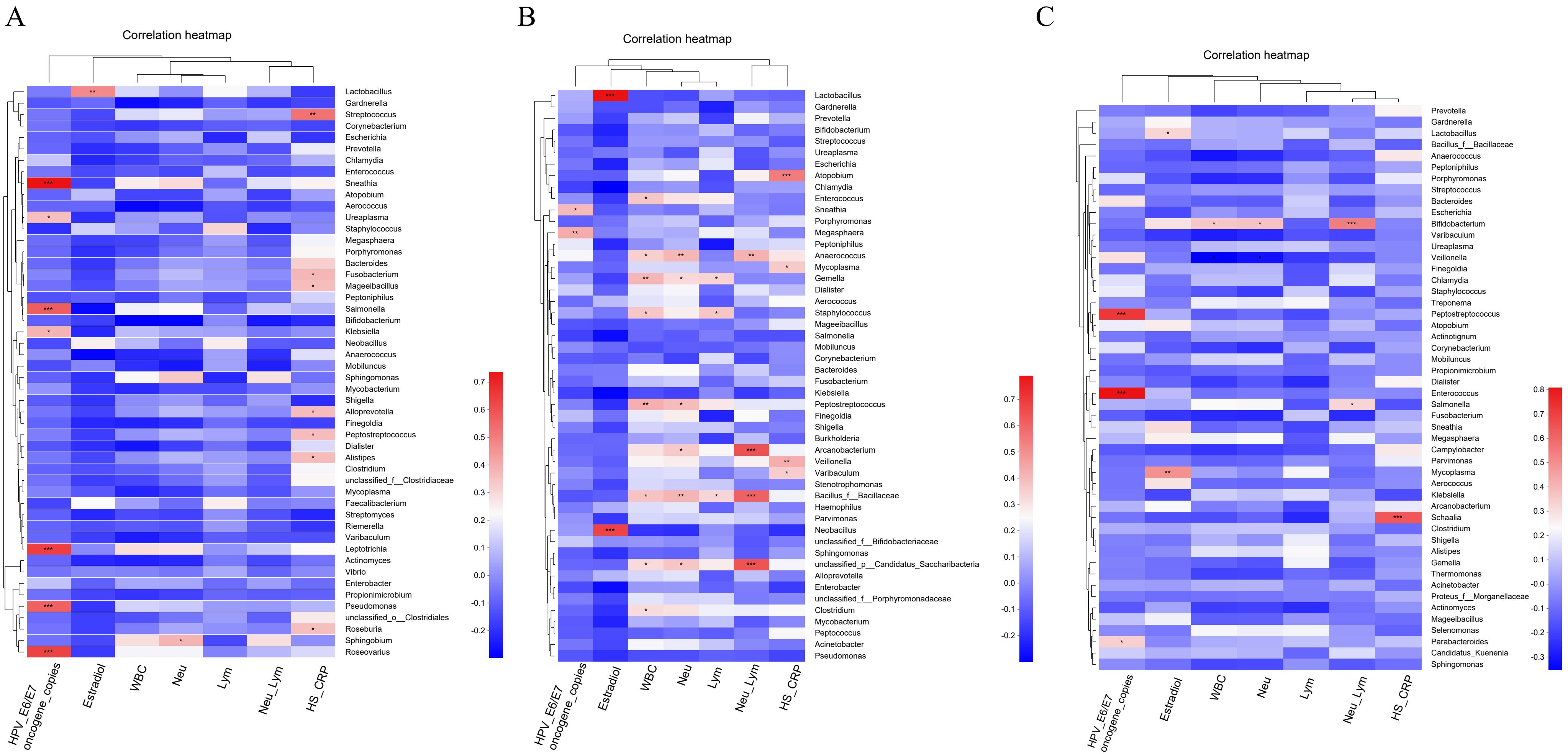

Figure S1. Correlation between microbiota genus, HPV oncogene expression, and blood indices. (A). HPV group; (B). CIN group; (C). CC group. Spearman's rank correlation coefficient is indicated using a color gradient: red indicates positive correlation; blue, negative correlation. *P < 0.05

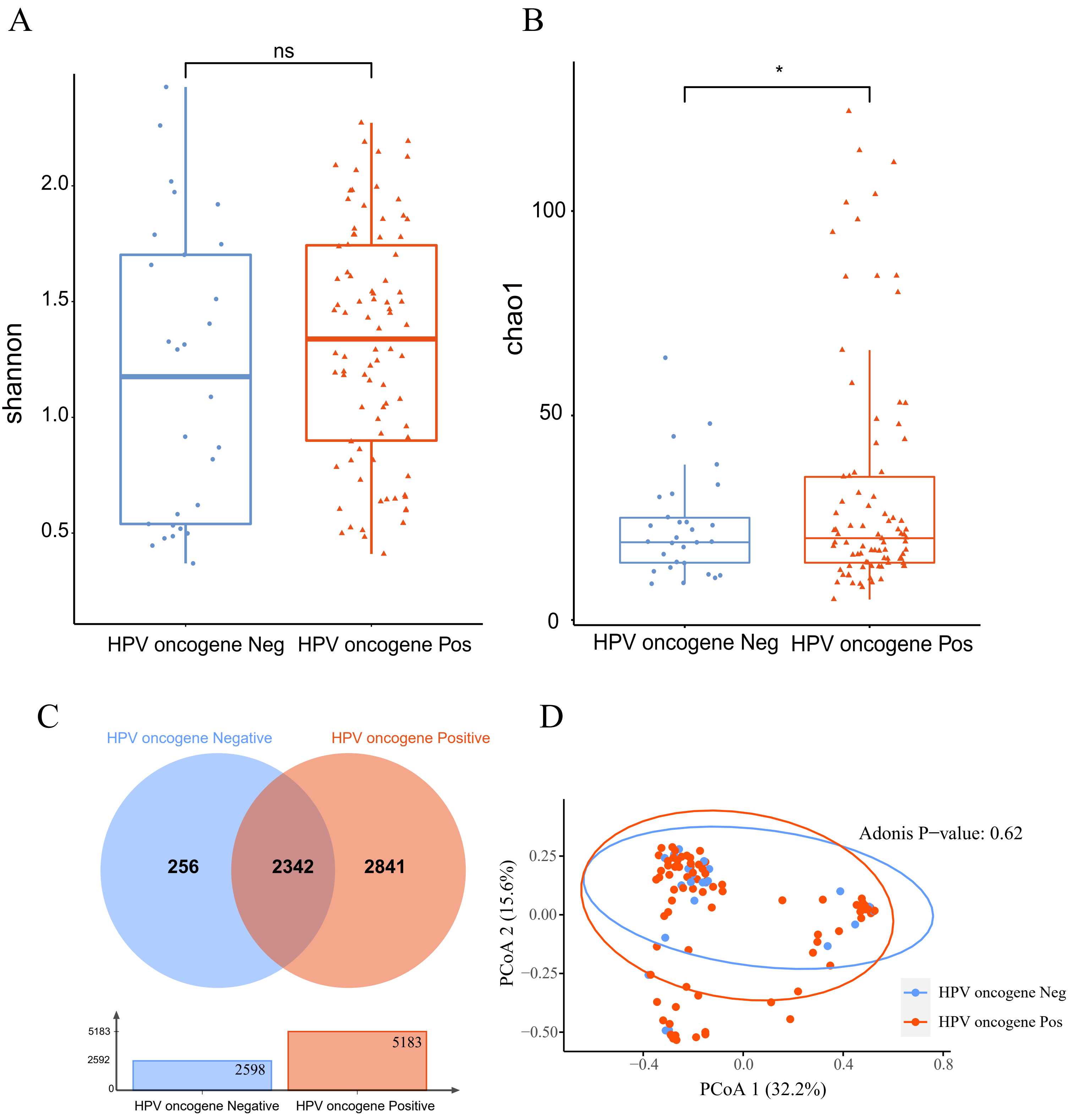

Figure S2. alpha-diversity and βeta-diversity between the HPV oncogene negative and positive group
(A). Shannon index (B). Chao1 index (C). Total species (D) Principal coordinate analysis between two groups. (Adonis analysis)

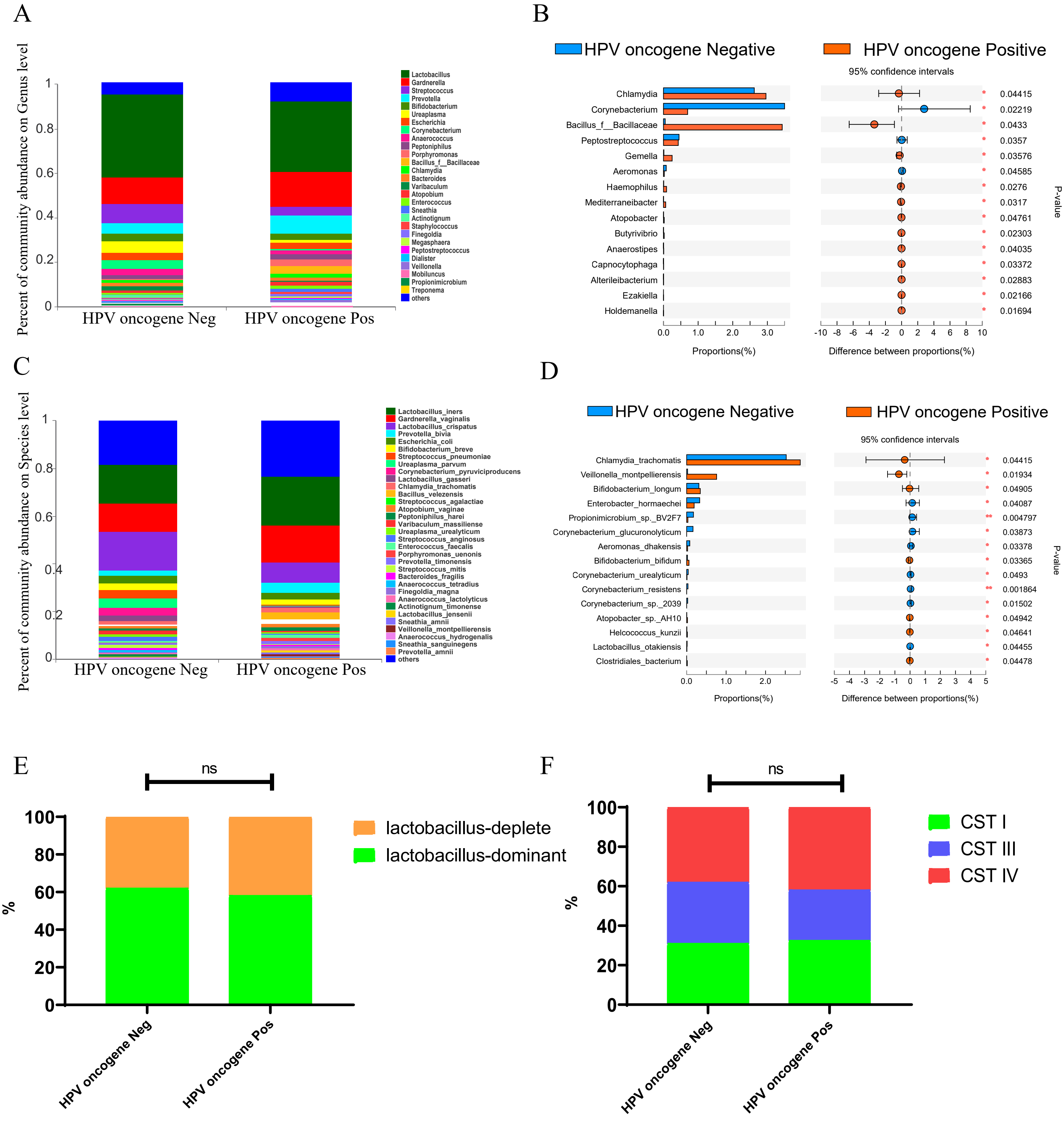

Figure S3. The different composition of genus and species of HPV oncogene negative and positive group.
(A) Histogram of microbial composition at the genus level; (B). Histogram of microbial composition at the species level;
(C). The significant different genus between two groups; (D). The significant different species between two groups;
(E). Lactobacillus composition between two groups; (F). composition of CST types between two groups.

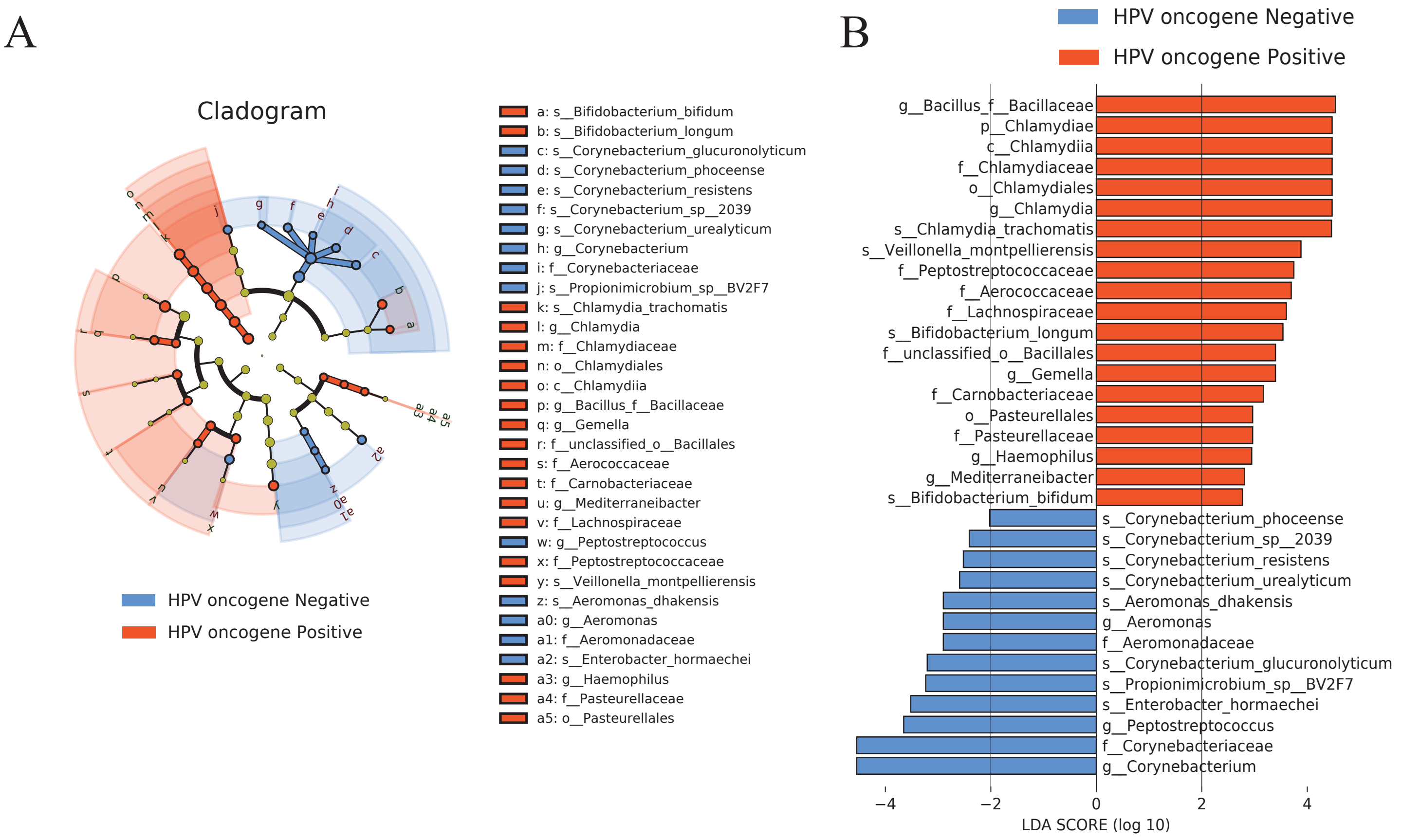

Figure S4. The LDA effect size (LEfSe) analysis to classify the underlying biomarkers uncovered the variety of the cervical microbiome based on the HPV oncogene group. LDA score>2.

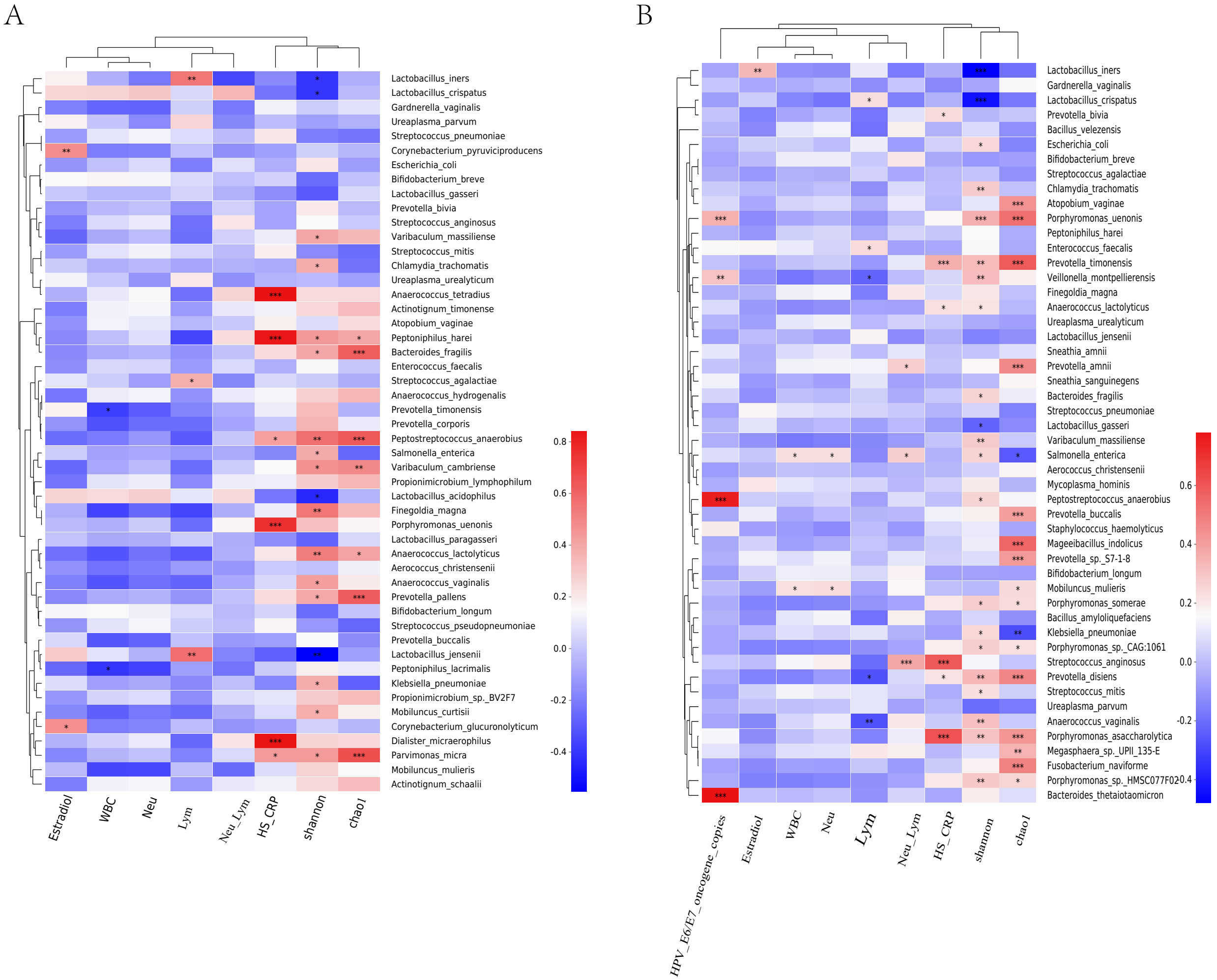

Figure S5. Correlation between microbiota genus, HPV oncogene expression, and blood indices. (A). HPV oncogene negative group; (B). HPV oncogene positive group. Spearman's rank correlation coefficient is indicated using a color gradient: red indicates positive correlation; blue, negative correlation. *P < 0.05. WBC white blood cell, Neu neutrophil, Lym Lymphocyte, HS-CRP High-Sensitivity C-reactive protein. Shannon index and Chao1 index alpha diversity of the cervical microbiome.

Table S1 Generated metagenomic data of each sample.

Samples	Group	HPV genotype	Clean reads	Clean base(bp)	Percent in raw reads(%)	Percent in raw bases(%)	Optimized reads	Optimized bases (bp)	Percent in raw reads (%)	Percent in raw bases (%)	Contigs	Contigs bases(bp)	N50(bp)	N90(bp)	Max(bp)	Min(bp)	ORFs	Total Length(bp)	Average Length(bp)	Max(bp)	Min(bp)
WDRM029	HPV	31+	68835906	10361372762	97.65744944	97.34882087	80048	11936248	0.113564039	0.11214534	330	169124	485	330	4529	300	372	129783	348.88	2247	105
WDRM030	HPV	43+,52+	79871522	12030143999	98.12818193	97.88038542	23258822	3510500985	28.57521504	28.5623505	13879	33176328	21762	645	370799	300	36655	29037855	792.19	10287	102
WDRM031	CIN	53+	67042854	10090948910	98.06547464	97.75043549	174154	26218141	0.254739971	0.253973608	119	1556104	41947	11106	223293	303	1261	1356198	1075.49	9633	111
WDRM032	HPV	52+	80676460	12144355532	97.96813098	97.6642265	160918	24149372	0.195408124	0.194207896	603	1535672	5012	1067	34891	300	1581	1327686	839.78	6168	102
WDRM033	HPV	33+,56+	81591566	12280247741	97.72389265	97.40599125	494552	74489090	0.592335077	0.590841797	2674	4474366	2604	638	283858	300	5818	3954306	679.67	8478	102
WDRM034	HPV	16+,39+	72349526	10889813177	96.93831815	96.62806267	113548	17014296	0.152138552	0.150972146	2052	1300639	668	370	16890	300	2569	1105179	430.2	2184	102
WDRM035	HPV	45+,81+	76084774	11454407328	97.96580739	97.67253122	21330	3161381	0.027464242	0.026957317	76	48145	540	320	7982	300	65	28629	440.45	1950	102
WDRM036	HPV	16+,52+,81+	77011696	11592742924	97.11345962	96.81262505	154422	23217809	0.194729573	0.193895185	1341	2095592	2295	683	18597	300	2948	1829133	620.47	5649	102
WDRM037	HPV	16+	74232990	11173028727	98.27656875	97.95959918	70952	10631756	0.093932888	0.093213987	953	1147919	1631	545	8046	301	1698	1009764	594.68	3888	102
WDRM038	HPV	31+	76925908	11578178342	98.36509362	98.04640312	99500	14936546	0.127230566	0.126485753	579	1345969	4133	954	36748	300	1669	1188771	712.27	4143	102
WDRM039	HPV	52+	84936144	12780182995	97.42292395	97.07977125	228818	34460065	0.262457389	0.261762702	4538	2384330	535	335	6246	300	5159	2014734	390.53	2190	102
WDRM040	HPV	52+	86687112	13037312186	97.63977133	97.24859529	249136	37503416	0.280613594	0.279747426	282	1470371	108502	4377	352204	300	1494	1270554	850.44	8724	102
WDRM041	HPV	39+	84914434	12783345990	98.25968516	97.96285765	2174046	327806760	2.515721597	2.512087758	408	3630234	33253	5446	251229	300	3733	3218124	862.07	8697	102
WDRM042	CIN	31+	81024376	12179766697	97.72916795	97.29052085	4241946	639425861	5.116507816	5.107657364	12627	20026703	6571	480	247819	300	25136	17846871	710.01	9597	102
WDRM043	HPV	53+	70598128	10624832938	97.34869185	97.02465282	84500	12706076	0.116518167	0.116030306	1903	1654257	1013	445	14893	300	2876	1438749	500.26	2604	102
WDRM044	HPV	31+	71934514	10817001620	98.07336715	97.66607166	11938	1753740	0.016275913	0.015834415	29	26698	3288	327	8076	302	24	16236	676.5	1890	114
WDRM045	HPV	16+	75064494	11295627327	97.45094698	97.11468603	1219068	183623112	1.582630145	1.578708322	83	1370813	127456	25699	256774	300	1324	1229121	928.34	9579	102
WDRM046	HPV	16+	73990448	11136052469	96.73550565	96.41943237	88658	13337298	0.115911941	0.115478506	1663	1592105	1312	421	19176	300	2574	1394256	541.67	4518	102
WDRM047	HPV	58+	79228870	11923095088	97.37402248	97.04467485	96144	14455455	0.118163089	0.117656105	2200	2187855	1238	480	12520	300	3405	1923261	564.83	8031	102
WDRM048	HPV	39+	70699708	10639741569	96.75232974	96.42684192	16280	2404145	0.022279129	0.021788509	37	16457	428	332	842	302	15	4623	308.2	768	102
WDRM049	CIN	68+	71026126	10692247298	97.04858381	96.75270647	5157120	778126849	7.046578783	7.041165109	25435	50036880	7588	573	222954	300	60564	44217879	730.1	10581	102
WDRM050	HPV	52+	77245024	11622518526	96.55333512	96.2099673	43714	6530839	0.054640833	0.054061588	297	135578	451	318	5062	300	314	110283	351.22	987	102
WDRM051	HPV	53+,58+	77209586	11616285124	95.61931168	95.27187345	32950	4851576	0.040806543	0.039790581	238	102292	423	318	1131	300	226	77271	341.91	828	102
WDRM052	HPV	59+	67505648	10160007766	97.32291249	97.00454419	355560	53619839	0.512610956	0.511945282	323	2271795	23874	3577	64487	302	2408	1988778	825.9	6756	102
WDRM053	HPV	58+	67807192	10204072755	96.81044281	96.48127762	488566	73680095	0.697540916	0.696658077	3638	7769062	5115	749	66593	300	9350	6835401	731.06	9549	102
WDRM054	CIN	52+	70913630	10673630211	96.70794425	96.39791479	2762004	416677312	3.766662754	3.763183024	21526	30640314	2957	499	225699	300	40232	26918781	669.09	11835	102
WDRM055	HPV	18+	72228848	10869097752	96.88792315	96.55516398	23838	3524769	0.031976342	0.031312134	34	16105	458	352	923	300	19	5724	301.26	753	108
WDRM056	HPV	51+	71055262	10691768927	96.95569121	96.6161391	264732	39797108	0.361229743	0.359626452	701	3376703	10729	2156	223197	301	3718	3019008	812	10218	102
WDRM057	HPV	58+	71036174	10689459851	96.70538957	96.37179088	35950	5372894	0.048940681	0.048439811	752	373212	505	336	4824	300	853	310737	364.29	2022	102
WDRM058	CIN	52+	73853126	11124665645	97.44197474	97.20468872	3255050	490837022	4.294720035	4.288817431	2961	8767915	17399	777	199502	300	8989	7594173	844.83	10011	102
WDRM059	HPV	16+	70674528	10634245087	97.00484642	96.66299199	20110	2973432	0.027602129	0.027027855	38	18297	503	319	1054	301	27	8121	300.78	732	102
WDRM060	HPV	52+	87554760	13173751838	96.07954229	95.73785533	27574	4095209	0.030258747	0.02976119	71	35702	533	346	1186	302	37	8895	240.41	606	102
WDRM061	HPV	16+	70198250	10566420575	96.74119838	96.43531934	1263146	190251422	1.740759318	1.736345483	4281	5233043	37990	383	194564	300	6968	4381683	628.83	12006	102
WDRM062	HPV	16+	81251432	12228193528	96.93692818	96.61478363	882646	132860349	1.053039803	1.049727733	3703	4869563	30076	433	362206	300	6792	4330971	637.66	9609	102
WDRM063	CIN	61+	70738202	10653704630	98.17604698	97.92086229	100890	15139871	0.140023087	0.139154339	431	210948	470	322	2188	300	482	173205	359.35	1824	102
WDRM064	CIN	52+	69684814	10494628712	98.29142709	98.03194662	48750	7327309	0.068762572	0.068445524	376	171626	429	323	8057	300	394	133917	339.89	1944	102
WDRM065	CIN	16+,52+	74206884	11170032771	98.01243153	97.70447777	15584	2290045	0.020583343	0.020031065	61	26725	425	317	879	302	34	8415	247.5	606	114
WDRM066	CIN	58+	69922644	10528915042	97.97187394	97.69902893	375904	56677408	0.526696606	0.52591627	1466	2961353	4937	672	159818	301	3276	2592321	791.31	9402	102
WDRM067	CIN	52+	68994200	10388721867	97.14064577	96.86649262	344074	51811948	0.484440294	0.483104827	2711	3969988	5576	453	40228	300	5351	3507669	655.52	9525	102
WDRM068	CIN	16+	68157672	10265627830	97.70448824	97.45594434	3311218	499590343	4.746653614	4.742822306	18864	29493164	4225	522	160897	300	37143	25829046	695.39	12993	102
WDRM069	CIN	52+	69304532	10437980017	97.95767504	97.70490067	1509730	227879613	2.133910099	2.133071238	5269	11229558	31303	528	244388	300	13297	9902553	744.72	15057	102
WDRM070	CIN	16+	47834434	7197053936	98.04615789	97.69396817	67100	10024668	0.137534756	0.136076456	405	176443	428	315	3129	300	423	144012	340.45	1881	102
WDRM071	CIN	52+	59754428	8986047257	97.07981195	96.68310376	338440	50890930	0.549845303	0.547548096	717	1578489	6743	655	41865	300	1965	1401537	713.25	8664	102
WDRM072	CIN	33+	77518130	11664634215	98.09949412	97.75920419	23048	3403916	0.029167333	0.028527609	65	31934	512	334	1204	301	40	12897	322.43	1113	105
WDRM073	CIN	33+	80806246	12163920047	98.33067139	98.02586488	1396454	210680276	1.699302544	1.697817495	868	2826798	11289	1085	57254	300	2883	2438103	845.68	6525	102
WDRM074	CIN	18+,58+	68612976	10326775314	97.80287064	97.48395173	20246	2990226	0.028859219	0.0282275	63	27974	433	326	966	301	37	10491	283.54	702	111
WDRM075	CIN	31+	83088594	12505283282	98.03870656	97.71759053	1248310	188318308	1.472918145	1.47153894	4687	5546880	14449	397	233756	300	7946	4843797	609.59	9828	102
WDRM076	CIN	16+	76143662	11459045030	98.2093051	97.87918787	164292	24721461	0.211902117	0.211162145	3494	2681948	870	383	11535	300	4668	2293623	491.35	3831	102
WDRM077	CIN	51+	81196738	12215757643	96.41983519	96.06634402	47412	7073524	0.056300996	0.055627134	395	186969	466	331	3118	300	426	145305	341.09	1731	102
WDRM078	HPV	31+	70970560	10678055103	96.02714858	95.68216895	546540	82333261	0.739499277	0.737758413	189	1359916	116157	8503	326071	302	1395	1219353	874.09	9189	102
WDRM079	CIN	58+	71882460	10818271565	96.5427282	96.22270639	1123686	169517929	1.509181963	1.507770794	1435	3450849	7293	808	236546	300	3841	3062307	797.27	9549	102
WDRM080	CIN	16+	66608770	10019688891	97.12411087	96.75489182	10312	1518477	0.015036216	0.014663138	25	16672	920	335	5120	303	19	8520	448.42	1935	120
WDRM081	CIN	35+	70411700	10596032707	97.80146884	97.46908526	137436	20630001	0.190897857	0.189767943	1601	2214865	2057	607	12432	300	2895	1912755	660.71	5079	102
WDRM082	HPV	52+	68290736	10276550166	96.39010049	96.05965866	252702	38018038	0.356680461	0.355372153	89	1361670	98177	21756	165995	302	1333	1223979	918.21	8832	102
WDRM083	CIN	58+	85199708	12817792076	96.54653449	96.19109272	101218	15215648	0.114698129	0.114185797	474	1338911	5482	1306	31758	300	1582	1191522	753.17	7116	102
WDRM084	CIN	58+	71939396	10824313716	96.88891317	96.54520756	282280	42492180	0.380178371	0.379000133	81	1363445	95335	27356	132540	307	1324	1220361	921.72	8943	102
WDRM085	CIN	16+	98529298	14826368659	97.23156963	96.89464046	1207616	182155927	1.191710502	1.190442074	2159	6775500	23889	754	164875	300	6743	5922918	878.38	10410	102
WDRM086	HPV	58+	76237382	11472762913	96.74898786	96.42053297	1315724	198514361	1.669718476	1.668374099	1016	2546603	15372	553	56427	300	3075	2230875	725.49	10488	102
WDRM087	CIN	58+	78061876	11742316276	97.76445884	97.39100401	450944	67988223	0.56476091	0.56389567	720	2327345	17874	1148	94672	300	2702	2043399	756.25	5664	102
WDRM088	CIN	39+,81+	76015800	11441121076	96.90770099	96.59308891	121168	18226904	0.154469364	0.153882906	139	1336344	37669	7921	186637	303	1322	1188438	898.97	8583	105
WDRM089	CIN	18+,81+	74028436	11140957520	97.28994915	96.96499363	17222	2544747	0.022633566	0.02214813	65	34492	497	332	4985	300	37	12120	327.57	1518	102
WDRM090	CIN	18+	72107132	10849154194	97.17442564	96.82615685	645740	97396723	0.870224787	0.869242911	2769	3628052	2102	482	429507	300	5034	3192150	634.12	9654	102
WDRM091	CIN	16+	72472926	10905104540	96.69978958	96.36129309	52664	7904729	0.070268968	0.069848932	1240	751092	631	359	7829	300	1551	656406	423.21	2169	102
WDRM092	CIN	18+,39+,59+	86570960	13027659366	97.41450787	97.08247835	30898	4590181	0.034768165	0.034206156	152	75209	489	338	1355	300	114	39810	349.21	1329	105
WDRM093	CIN	58+	77856788	11713491500	97.10720591	96.75298995	494410	74492897	0.61665495	0.615308468	1717	4749530	8983	940	366067	300	5159	4167093	807.73	9612	102
WDRM094	CIN	16+,18+	70164504	10556200149	97.97623637	97.61891173	360474	53913063	0.503358305	0.498563353	2917	4768955	4003	526	47075	300	6213	4147323	667.52	5103	102
WDRM095	CIN	16+,52+	71020994	10686688433	97.21521324	96.87543984	177186	26618300	0.24253638	0.241296407	108	1614591	42703	13420	190423	302	1314	1409538	1072.71	9597	102
WDRM096	HPV	16+,51+	85101712	12798907698	95.47342772	95.09116714	407500	61399556	0.457163797	0.456176072	436	2252318	17413	2537	83327	300	2448	1973841	806.31	6474	102
WDRM097	CIN	52+	91210856	13710756690	97.9748327	97.53320052	61286	9146567	0.065830822	0.06506526	808	536536	712	366	3943	300	998	475896	476.85	3096	102
WDRM098	CIN	16+,39+	70697824	10637527319	97.81873285	97.47196624	117874	17686662	0.163092506	0.162063388	425	1451821	7055	1568	75149	303	1648	1300761	789.3	6252	102
WDRM099	CIN	58+,68+	71613058	10774679847	97.87293648	97.52092831	58672	8672911	0.080186506	0.078497955	66	30762	485	325	1082	305	42	10587	252.07	723	102
WDRM100	HPV	16+	67110290	10099309200	98.17639919	97.84366351	1481818	223624396	2.167768244	2.166507602	311	2261022	19316	3909	84263	300	2412	1980912	821.27	6762	102
WDRM101	CIN	33+,52+	69186114	10417142314	98.10628773	97.82493197	42494	6343455	0.060256724	0.059569893	354	165925	460	328	3401	300	371	137739	371.26	996	102
WDRM102	CIN	58+,81+	72415960	10897923807	97.65508721	97.32566946	919128	138489796	1.239471589	1.236805501	250	1624142	76362	12060	250455	300	1378	1403523	1018.52	9573	102
WDRM103	CC	16+	67541390	10169768000	97.98311835	97.70467874	81630	12226220	0.118421637	0.117461765	152	69387	428	317	4851	300	137	49749	363.13	1950	102
WDRM104	CC	16+	72527624	10914352915	98.08638858	97.75215483	38594	5726485	0.052194542	0.051288084	60	35250	545	327	7914	300	50	20199	403.98	1950	105
WDRM105	CC	16+	70278420	10585942318	97.57399873	97.33402664	36916	5525135	0.05125388	0.050801678	55	31780	548	330	3622	305	36	13719	381.08	1167	102
WDRM106	CC	58+	67080922	10095674364	97.03027786	96.70894343	68168	10157005	0.098602699	0.097296445	83	36606	458	321	750	302	68	18912	278.12	678	111
WDRM107	CC	45+	47778662	7186974340	98.15943643	97.78386962	27246	4022474	0.055975866	0.054728604	37	15329	450	310	558	300	24	6177	257.38	489	102

WDRM108	CC	52+	57359488	8629389400	98.16771138	97.80623015	48242	7107938	0.082563616	0.080561971	45	20013	471	326	793	303	26	7650	294.23	603	117
WDRM109	CC	16+	54805430	8245986730	98.16104898	97.80955556	79814	11937073	0.142953462	0.141591279	1291	620991	474	327	4232	300	1427	524577	367.61	1599	102
WDRM110	CC	16+	48119492	7239962068	98.11398143	97.76180451	1656038	249793736	3.376604258	3.372985405	33450	27168745	909	390	66901	300	46284	24006333	518.67	10332	102
WDRM111	CC	16+	50815060	7644843549	98.07817193	97.71732608	67780	10115514	0.130822211	0.129297738	695	352804	511	338	7583	300	820	306336	373.58	2145	102
WDRM112	CC	45+	50956740	7668026785	98.48792043	98.14948055	3286302	495828244	6.351682817	6.346519901	34097	45182276	2163	495	222642	300	63345	39982362	631.18	14421	102
WDRM113	CC	16+	76047312	11438681084	98.12154279	97.74161366	598238	90118976	0.771888368	0.770051553	10483	9259874	1109	401	29176	300	14732	8051421	546.53	4704	102
WDRM114	CC	33+	49730372	7482209590	98.06985843	97.71624338	20130	2955753	0.039696993	0.038601576	25	18123	640	331	7928	300	24	11343	472.63	1935	105
WDRM115	CC	52+	63531438	9554132305	98.05985415	97.66003177	26388	3767343	0.040729496	0.03850887	25	10560	428	309	792	304	10	3153	315.3	792	126
WDRM116	CC	16+	46096474	6933761230	97.91774832	97.54065022	71426	10630474	0.151722518	0.149544138	154	128194	991	405	4890	305	190	99753	525.02	3339	102
WDRM117	CC	66+	71293314	10729272061	96.44145804	96.1187856	1032714	155754616	1.396995571	1.395336464	10115	15164050	3150	527	164975	300	19409	13375752	689.15	10287	102
WDRM118	CC	45+	67633990	10174020496	96.86960207	96.50241186	59900	8999434	0.085792501	0.085361248	517	235894	448	318	5443	300	533	189774	356.05	1911	102
WDRM119	CC	52+	69461726	10447719194	96.87912207	96.50041213	49856	7423612	0.069534775	0.06856823	125	60798	490	322	1561	300	98	29931	305.42	861	111
WDRM121	CC	33+	71731956	10790079612	97.68281918	97.30904568	101042	15095393	0.137596519	0.136136001	676	1226787	3042	722	37043	301	1601	1101390	687.94	7278	102
WDRM122	CC	16+	73949798	11102961875	95.98574947	95.44027225	20970	3099015	0.027218751	0.026638913	53	33254	631	374	7921	309	41	16173	394.46	1935	102
WDRM123	CC	58+	75808086	11407281920	96.41961931	96.08489244	27612	4106110	0.035119453	0.034586253	73	34463	490	333	1042	304	48	14328	298.5	630	105
WDRM124	CC	18+	71545152	10763740771	95.93262105	95.58117877	543344	81949770	0.728552705	0.727707568	188	2277297	45659	9892	127682	302	1959	1968891	1005.05	5565	102
WDRM125	CC	16+	72791852	10954616290	96.7434066	96.41815786	1535506	231599557	2.040751502	2.038446812	1235	5420757	39645	1796	319605	300	5535	4792221	865.8	9846	102
WDRM126	CC	45+,81+	67314702	10132028766	97.09984424	96.78946126	30286	4531005	0.043686829	0.043283882	65	30694	451	337	1370	300	37	10740	290.27	534	102
WDRM127	CC	18+	75939010	11424157699	96.7126097	96.35303826	466846	70381546	0.594554696	0.593608384	9292	8422262	1129	399	54285	300	13273	7300149	550	9909	102
WDRM128	CC	52+	71528558	10758134889	96.48211535	96.1008845	82012	12247053	0.110622826	0.109401178	59	26568	453	328	934	301	34	11709	344.38	879	132
WDRM129	CC	39+	71751752	10784451384	97.41423717	96.96411407	197240	29608316	0.267784181	0.266211421	1139	481383	411	317	7979	300	1184	409125	345.54	2055	102
WDRM130	CC	16+	75989496	11433729082	96.78947722	96.4462909	40778	6070573	0.051939827	0.051206762	35	23567	605	337	7946	301	28	11514	411.21	1950	111
WDRM131	CC	18+	75321920	11336193483	97.21665436	96.89683499	13880	2057057	0.017914668	0.017582826	39	17041	448	317	928	300	19	4269	224.68	510	111
WDRM132	CC	16+,35+,52+,58+	69576288	10466748002	96.42722105	96.06677623	197156	29656231	0.273242591	0.272193284	1883	2329133	5027	400	103333	300	3150	2023356	642.34	9609	102
WDRM133	CC	52+,82+	95382480	14351955784	97.752435	97.40766093	55872	8310294	0.057260244	0.056402508	198	99633	516	324	1532	301	152	54654	359.57	1389	102
WDRM135	CC	16+	84206272	12671587276	97.60051136	97.26614965	47078	7014218	0.054566445	0.053840609	181	86688	471	330	2104	300	165	54264	328.87	1221	105
WDRM136	CC	45+	76385090	11480117819	97.76572715	97.30775164	9564454	1442959299	12.24160108	12.23080872	25108	33679051	5784	437	313329	300	46118	29718834	644.41	10605	102
WDRM137	CC	58+	88369942	13297671068	98.12650975	97.78684191	37986	5633035	0.042179881	0.041423547	116	50446	416	325	811	305	83	24603	296.42	777	102
WDRM138	CC	52+	72881146	10966414976	97.97256704	97.62859133	95824	14339934	0.128814155	0.12766137	279	121697	428	317	1738	301	264	87720	332.27	1335	102
WDRM140	CC	16+	70287524	10575708213	97.96463599	97.616582	59892	8941884	0.083475667	0.082535953	356	159882	459	314	1544	300	364	120069	329.86	1203	102
WDRM141	CC	16+	71255798	10717359351	96.53456511	96.15535636	906290	136625737	1.227806206	1.225796019	8733	9989874	2299	407	168923	300	14647	8800827	600.86	9909	102
WDRM142	CC	16+	71283992	10725878396	97.88843886	97.54282092	46402	6857764	0.063720047	0.062365582	83	43187	483	337	2460	302	59	19155	324.66	1098	102
WDRM143	CC	56+	71395016	10749669594	97.85204965	97.57087513	6189218	933927013	8.482772342	8.476918771	16760	24083399	4099	476	259594	300	32538	21265587	653.56	11049	102
WDRM144	CC	16+,61+	70712096	10648430867	98.76258107	98.49345887	16251518	2451412816	22.69826458	22.67452645	18375	33624581	6547	540	209588	300	39930	29603736	741.39	22956	102
WDRM145	CC	58+	71757588	10795880254	97.7921348	97.4354973	330676	49705021	0.450649372	0.448600144	6945	5980475	1028	428	13536	300	9931	5299416	533.62	5919	102
WDRM146	CC	51+,53+,56+	72921492	10970512862	98.05026756	97.68845047	1951964	294260203	2.624611582	2.620280713	1657	4298502	5337	949	194506	300	4688	3809985	812.71	8934	102

Table S2 Distribution of the samples of the three groups in the vaginal community state type (CST).

Type	HPV (%)	CIN (%)	CC (%)
CST I	9 (26.47)	3 (7.50)	0 (0.00)
CST III	14 (41.18)	8 (20.00)	4 (9.76)
CST IV	11 (32.35)	29 (72.50)	37 (90.24)
Total	34	40	41
Type	HPV (%)	CIN (%)	CC (%)
Lactobacillus -dominate	23 (67.65)	11 (27.50)	4 (9.76)
Lactobacillus -deplete	11 (32.35)	29 (72.50)	37 (90.24)
Total	34	40	41

Table S3 Relative abundance of the different Genus among three groups

Genus	HPV-Mean(%)	HPV-Sd(%)	CIN-Mean(%)	CIN-Sd(%)	CC-Mean(%)	CC-Sd(%)	P-value	Corrected p-value
g__Lactobacillus	62.1	41.59	25.71	37.41	9.193	21.23	1.33E-6	0.001711
g__Prevotella	2.511	7.571	6.714	11.96	11.51	19.89	0.0001935	0.06441
g__Escherichia	4.535	14.09	3.162	8.107	4.32	10.93	0.01236	0.5505
g__Bifidobacterium	0.2204	0.4472	5.892	21.17	2.772	10.33	0.0059	0.4427
g__Bacillus_f__Bacillaceae	0.03947	0.1469	0.1416	0.4153	7.055	19.31	0.0007192	0.1028
g__Porphyromonas	0.3051	0.9367	1.377	3.777	4.091	9.304	0.01148	0.5505
g__Anaerococcus	0.1692	0.3471	0.7691	1.506	4.166	7.617	0.01241	0.5505
g__Bacteroides	0.2706	0.9167	0.365	0.8954	3.037	6.514	1.6E-5	0.01029
g__Veillonella	0.0252	0.06526	0.1985	0.7517	1.549	4.404	0.01295	0.5552
g__Dialister	0.08766	0.2156	0.5691	0.9624	0.6303	0.9533	0.003093	0.2841
g__Treponema	0.004661	0.017	0.01111	0.02329	0.9916	5.742	0.0002517	0.06441
g__Campylobacter	0.01102	0.03196	0.08333	0.2573	0.4088	0.9152	0.0003742	0.06441
g__Alistipes	0.06229	0.3458	0.04601	0.1692	0.3023	1.126	0.01056	0.5505
g__Acinetobacter	0.02962	0.1513	0.1778	0.8164	0.1679	0.5154	0.009373	0.5479
g__Neobacillus	0.1861	0.174	0.1077	0.1829	0.0569	0.1487	0.0004007	0.06441
g__Stenotrophomonas	0	0	0.132	0.8278	0.1065	0.6025	0.01663	0.5995
g__Parabacteroides	0.01491	0.06585	0.03328	0.06593	0.1792	0.619	0.0003109	0.06441
g__Schaalia	0.001117	0.003453	0.016	0.05779	0.1726	0.5651	0.04032	0.5995
g__Trichococcus	0.009819	0.01081	0.1457	0.8715	0.003962	0.008825	0.005935	0.4427
g__Candidatus_Kuenenia	0	0	0.03195	0.1664	0.1058	0.3979	0.02946	0.5995
g__Microbacterium	0	0	0.02193	0.08582	0.1024	0.3369	0.008778	0.5479
g__unclassified_f__Porphyromonadaceae	0.01027	0.04677	0.07157	0.3751	0.04027	0.16	0.02781	0.5995
g__Clostridioides	0.01646	0.05569	0.05577	0.191	0.04534	0.09855	0.01758	0.5995
g__Tannerella	0.01379	0.04778	0.03358	0.08271	0.06579	0.1114	0.001702	0.2161
g__Phascolarctobacterium	0.01756	0.01785	0.08531	0.3486	0.009805	0.01675	0.01089	0.5505
g__Leptotrichia	0.05346	0.1347	0.03782	0.07039	0.01697	0.03928	0.001848	0.2161
g__Butyricimonas	0.002759	0.01471	0.006787	0.01562	0.0853	0.4309	0.03286	0.5995
g__Facklamia	0.001916	0.006379	0.01544	0.06242	0.05101	0.09028	0.00228	0.2443
g__Odoribacter	0.01457	0.06287	0.01006	0.02792	0.03215	0.06181	0.03015	0.5995
g__Peptoanaerobacter	0.003764	0.01754	0.006497	0.01585	0.03644	0.1024	0.005884	0.4427
g__Eggerthella	0.005658	0.02282	0.01644	0.03467	0.02427	0.07959	0.0338	0.5995
g__unclassified_o__Bacteroidales	0.01283	0.06926	0.009375	0.03108	0.02405	0.05103	0.0169	0.5995
g__Neisseria	0.001315	0.003964	0.02086	0.06356	0.02385	0.1196	0.033	0.5995
g__Urinicoccus	0.0005838	0.002102	0.004696	0.009715	0.02877	0.05808	0.006541	0.4427
g__Phocaeicola	0.001897	0.009772	0.002569	0.005496	0.02648	0.1217	0.02035	0.5995
g__Lawsonella	0	0	0.0004414	0.002009	0.02731	0.08475	0.006381	0.4427
g__Enterocloster	0.003662	0.01585	0.007528	0.02062	0.01646	0.03424	0.0343	0.5995
g__Paraprevotella	0.001939	0.007939	0.009721	0.02479	0.01344	0.03483	0.01038	0.5505
g__Dorea	0.002652	0.01325	0.006922	0.0168	0.01161	0.02404	0.01454	0.5995
g__Coprococcus	0.00286	0.01324	0.005469	0.01047	0.01213	0.03523	0.03861	0.5995
g__Trueperella	0.002763	0.0161	0.00136	0.006106	0.01309	0.03972	0.04237	0.5995
g__Syntrophomonas	0.0006628	0.002538	0.001069	0.002335	0.0153	0.04909	0.02329	0.5995
g__Lupinus	0.002484	0.009076	0.007377	0.01249	0.006343	0.01293	0.02847	0.5995
g__Metaprevotella	0.001395	0.005669	0.005136	0.01537	0.009168	0.02011	0.03294	0.5995
g__unclassified_d__Bacteria	0.001807	0.01009	0.003883	0.01009	0.009267	0.01987	0.03423	0.5995
g__Pseudoglutamicibacter	7.441E-6	4.339E-5	7.874E-5	0.000498	0.01361	0.05153	0.01935	0.5995
g__Pseudoflavonifractor	0.001249	0.005093	0.004407	0.01325	0.005447	0.0153	0.04896	0.5995
g__Caldalkalibacillus	0.002056	0.006273	0.005197	0.01008	0.002371	0.008859	0.04948	0.5995
g__Epulopiscium	0.0009109	0.003964	0.0002044	0.0007879	0.008358	0.02314	0.00254	0.2512
g__Dermabacter	0.004131	0.02409	0.0008335	0.004446	0.002621	0.008492	0.0229	0.5995
g__Microscilla	1.284E-5	7.489E-5	0.0001002	0.0004656	0.007171	0.04063	0.04984	0.5995
g__Ornithobacterium	0.001174	0.005005	0.001845	0.005033	0.003481	0.006478	0.04983	0.5995
g__Coprobacillus	0.0008354	0.003392	0.003042	0.006346	0.002535	0.007041	0.04267	0.5995
g__Ezakiella	0	0	0.0001345	0.0004745	0.005784	0.02338	0.01185	0.5505
g__Allisonella	3.587E-5	0.0002092	0.002537	0.007249	0.002996	0.01025	0.03169	0.5995
g__Gleimia	0.0001307	0.0007619	0.0009609	0.002789	0.00398	0.01446	0.02207	0.5995
g__Limnohabitans	0.003877	0.00868	0.0004086	0.002584	0	0	0.0002752	0.06441
g__Jeotgalibaca	0.0008852	0.003591	0.002402	0.005825	0.0003862	0.001187	0.03336	0.5995
g__Pseudoscardovia	0	0	0.002071	0.008356	0.0001435	0.0008638	0.03252	0.5995
g__Keratinibaculum	0	0	0	0	0.002067	0.01142	0.009341	0.5479
g__Hydrogenimonas	0	0	0	0	0.001581	0.008569	0.02458	0.5995
g__Daphnia	0.000976	0.004312	0.0001886	0.001193	0	0	0.03639	0.5995
g__Saccharibacillus	3.847E-5	0.0002243	0	0	0.0009838	0.004253	0.03636	0.5995
g__Pseudopropionibacterium	0	0	0	0	0.0007173	0.003108	0.02458	0.5995
g__Clostridiisalibacter	0	0	3.164E-5	0.0002001	0.0006154	0.003128	0.01668	0.5995
g__Propioniferax	0	0	0	0	0.0005633	0.002389	0.02458	0.5995
g__Gracilibacillus	0	0	0	0	0.0004529	0.002163	0.02458	0.5995
g__Helcobacillus	0	0	0	0	0.0004156	0.002319	0.02458	0.5995
g__Xanthobacter	0	0	0.0001324	0.0004363	1.49E-5	9.542E-5	0.03386	0.5995

Table S4 Relative abundance of the different Species among three groups

Species	HPV-Mean(%)	HPV-Sd(%)	CIN-Mean(%)	CIN-Sd(%)	CC-Mean(%)	CC-Sd(%)	P-value	Corrected p-value
s__Lactobacillus_iners	32.19	36.36	17.15	32.26	7.246	20.05	0.001096	0.1591
s__Lactobacillus_crispatus	23.63	37.08	6.209	21.27	0.8459	2.446	0.0001403	0.06936
s__Escherichia_coli	4.456	13.83	3.103	7.872	4.202	10.65	0.0145	0.5838
s__Prevotella_bivia	0.62	2.007	2.92	7.974	7.117	18.05	0.003559	0.2978
s__Bacillus_velezensis	0	0	0.06444	0.336	5.981	16.84	0.0009672	0.1591
s__Lactobacillus_gasseri	2.392	7.72	0.4957	1.166	0.3889	1.279	5.824E-5	0.06335
s__Lactobacillus_jensenii	1.213	3.273	0.7376	2.901	0.1981	0.4617	0.0007058	0.1591
s__Streptococcus_mitis	0.7526	3.898	0.6944	1.684	0.5174	1.993	0.0479	0.5902
s__Anaerococcus_lactolyticus	0.01665	0.03664	0.2598	0.8354	1.495	5.029	0.008049	0.4467
s__Veillonella_montpellierensis	0.01326	0.05039	0.1714	0.6955	1.43	4.109	0.0005648	0.1591
s__Bacteroides_fragilis	0.05766	0.1682	0.1316	0.5479	1.347	3.818	9.105E-5	0.06591
s__Prevotella_amnii	0.05319	0.1617	0.6093	2.161	0.5352	1.431	0.01722	0.5902
s__Bifidobacterium_longum	0.08557	0.2222	0.5965	1.846	0.2905	0.9562	0.01252	0.5448
s__Anaerococcus_tetradius	0.04519	0.2061	0.1581	0.4655	0.7488	3.806	0.009163	0.4652
s__Lactobacillus_acidophilus	0.5947	0.9197	0.1349	0.4289	0.0139	0.03713	0.0008387	0.1591
s__Anaerococcus_vaginalis	0.004955	0.01172	0.1417	0.3801	0.5383	1.266	0.009981	0.4891
s__Lactobacillus_paragasseri	0.3215	1.127	0.2146	0.8908	0.08993	0.5127	0.00238	0.2467
s__Bacillus_amyloliquefaciens	0	0	0.005239	0.02575	0.597	1.729	0.0009672	0.1591
s__Anaerococcus_hydrogenalis	0.02103	0.07713	0.0276	0.05613	0.5369	1.92	0.01651	0.5902
s__Dialister_micraerophilus	0.04605	0.1167	0.1939	0.3	0.3032	0.6709	0.02281	0.5902
s__Staphylococcus_epidermidis	0.2248	0.2162	0.1209	0.1994	0.1616	0.542	0.005522	0.3762
s__Lactobacillus_farciminis	0.2489	0.2838	0.1359	0.2701	0.08137	0.2363	0.009065	0.4652
s__Lactobacillus_paracasei	0.2567	0.2914	0.1384	0.2685	0.05823	0.1851	0.0006458	0.1591
s__Bacteroides_thetaiotaomicron	0.01138	0.04374	0.009567	0.02042	0.3695	1.93	0.00188	0.2175
s__Porphyromonas_sp._HMSC077F02	0.004876	0.01727	0.04587	0.1888	0.3388	1.044	0.02635	0.5902
s__Bacteroides_ovatus	0.01054	0.04799	0.0302	0.07291	0.3385	1.014	1.361E-5	0.02468
s__Fusobacterium_nucleatum	0.02435	0.08665	0.126	0.6273	0.2011	0.6365	0.01715	0.5902
s__Neobacillus_vireti	0.1855	0.1746	0.1075	0.183	0.05629	0.1488	0.001001	0.1591
s__Arcanobacterium_sp._S3PF19	0.003978	0.02256	0.1586	0.9656	0.1841	0.7162	0.04336	0.5902
s__Lactobacillus_helveticus	0.2186	0.3225	0.06451	0.2067	0.03062	0.08983	0.001337	0.1818
s__Acinetobacter_baumannii	0.02954	0.1513	0.1777	0.8164	0.0865	0.2359	0.03485	0.5902
s__Prevotella_colorans	0.07655	0.319	0.1203	0.2404	0.07814	0.1622	0.01244	0.5448
s__Prevotella_pallens	0.006956	0.02769	0.02511	0.05262	0.2379	1.226	0.0001126	0.06591
s__Corynebacterium_striatum	0.0683	0.3292	0.03195	0.06813	0.1612	0.5055	0.04681	0.5902
s__Stenotrophomonas_maltophilia	0	0	0.1319	0.8278	0.1028	0.6025	0.01663	0.5902
s__Parvimonas_micra	0.01135	0.04684	0.02945	0.07865	0.1845	0.6402	0.03821	0.5902
s__Lactobacillus_amylovorus	0.166	0.2697	0.04602	0.1596	0.009578	0.04535	0.01337	0.5595
s__Peptoniphilus_sp._Marseille-P3761	0.00158	0.006232	0.01386	0.06554	0.202	1.094	0.03033	0.5902
s__Dialister_invisus	0.01011	0.0351	0.1117	0.2823	0.08544	0.2056	0.03695	0.5902
s__Anaerococcus_sp._Marseille-P3915	0.001373	0.005282	0.01978	0.09656	0.1838	0.4356	0.002477	0.2467
s__Prevotella_intermedia	0.01886	0.06988	0.0867	0.1842	0.0974	0.2364	0.001606	0.2032
s__Bifidobacterium_dentium	7.829E-5	0.0004095	0.05733	0.2435	0.1378	0.6059	0.0279	0.5902
s__Lactobacillus_johnsonii	0.131	0.234	0.04351	0.08944	0.01579	0.0538	0.0001132	0.06591
s__Streptococcus_sp._UMB1385	7.981E-5	0.0004654	0.1815	1.095	0.006758	0.03763	0.02283	0.5902
s__Schaalia_turicensis	0.001107	0.003443	0.01429	0.05707	0.1654	0.5515	0.04545	0.5902
s__Bacteroides_xylanisolvens	0.02288	0.04354	0.0286	0.05647	0.1291	0.2153	0.0008243	0.1591
s__Treponema_denticola	0	0	0.0005071	0.003207	0.1667	0.9985	0.002469	0.2467
s__Prevotella_bergensis	0.01676	0.06679	0.05309	0.1465	0.09347	0.2544	0.001828	0.2161
s__Bifidobacterium_adolescentis	0.02315	0.05805	0.08578	0.1004	0.04982	0.09524	0.01311	0.5595
s__Trichococcus_palustris	0.009819	0.01081	0.1455	0.8715	0.003416	0.008788	0.001443	0.1914
s__Prevotella_stercorea	0.001735	0.008168	0.006533	0.01953	0.1497	0.8309	0.02037	0.5902
s__Bacteroides_salyersiae	0.009887	0.04542	0.006267	0.02045	0.138	0.6377	0.002752	0.2581
s__Bacteroides_uniformis	0.01194	0.04798	0.01742	0.0327	0.1191	0.2708	9.498E-6	0.02468
s__unclassified_g__Bacillus_f__Bacillace	0	0	0.002149	0.009805	0.1446	0.4084	0.0009672	0.1591
s__Streptococcus_sp._Z2	0.1397	0.7855	0.0003203	0.0009616	0.001268	0.006925	0.0143	0.5838
s__Escherichia_fergusonii	0.04282	0.2461	0.0528	0.3038	0.04512	0.18	0.04165	0.5902
s__Bifidobacterium_bifidum	0.0001504	0.0008769	0.06593	0.1851	0.07291	0.3772	0.006693	0.42
s__Candidatus_Kuenenia_stuttgartiensis	0	0	0.03195	0.1664	0.1058	0.3979	0.02946	0.5902
s__Campylobacter_showae	0.0004397	0.001713	0.0001243	0.0004676	0.1335	0.6656	0.03206	0.5902
s__Roseovarius_nitratireducens	0.04894	0.2028	0.06087	0.2158	0.02182	0.1397	0.02766	0.5902
s__Bifidobacterium_pseudocatenulatum	0.01382	0.03828	0.0786	0.1425	0.03036	0.06455	0.007594	0.4414
s__Lactobacillus_amylolyticus	0.08643	0.1373	0.03019	0.09704	0.003969	0.01448	0.04167	0.5902
s__Clostridioides_difficile	0.01576	0.05417	0.05531	0.1911	0.04499	0.09852	0.01592	0.5902
s__Campylobacter_sp._AAUH-44UCsig-a	0.0004935	0.00164	0.02388	0.1399	0.08776	0.3185	0.01946	0.5902
s__uncultured_Faecalibacterium_sp.	0.04677	0.04331	0.02163	0.03628	0.03308	0.06377	0.03526	0.5902
s__Phascolarctobacterium_sp._CAG:207	0.01728	0.01764	0.07496	0.348	0.008397	0.01485	0.01457	0.5838
s__Treponema_medium	0	0	0.0008361	0.005288	0.09561	0.4773	0.03923	0.5902
s__Anaerococcus_obesiensis	0.007038	0.02618	0.03518	0.1154	0.05392	0.1345	0.04383	0.5902
s__Enterococcus_faecium	0.009358	0.03232	0.01465	0.03663	0.07079	0.2252	0.01266	0.5465
s__Dialister_invisus_CAG:218	0.005039	0.01761	0.03579	0.08621	0.05165	0.09685	0.04511	0.5902
s__Varibaculum_vaginae	6.144E-5	0.0002787	0.01426	0.08324	0.07794	0.2676	0.0152	0.589
s__Leptotrichia_hofstadii	0.05328	0.1348	0.02771	0.05364	0.01086	0.02977	0.0001834	0.08311
s__Lactobacillus_gallinarum	0.06145	0.07235	0.01885	0.03727	0.01083	0.03966	0.000451	0.1398
s__Anaerococcus_sp._HMSC075B03	0.01013	0.05188	0.01262	0.04312	0.06711	0.1533	0.04786	0.5902
s__Prevotella_copri	0.01207	0.05863	0.02377	0.06052	0.05238	0.1289	0.00364	0.2999
s__Bacteroides_stercoris	0.01022	0.04098	0.00815	0.02218	0.06968	0.3338	0.006936	0.42
s__Peptoniphilus_duerdenii	0.001923	0.004532	0.02557	0.1128	0.0603	0.1456	0.03396	0.5902
s__Prevotella_melaninogenica	0.02471	0.1364	0.02353	0.05726	0.03821	0.1084	0.007632	0.4414
s__Streptococcus_equi	0.001159	0.00405	0.009799	0.03164	0.07412	0.3231	0.01826	0.5902

s__Anaerococcus_mediterraneensis	0.0005205	0.001507	0.002307	0.005529	0.08022	0.2203	0.003514	0.2978
s__Peptoniphilus_sp._BV3AC2	0.0009035	0.004213	0.007352	0.01946	0.07382	0.2139	0.01319	0.5595
s__Streptococcus_dysgalactiae	0.01555	0.0623	0.01721	0.03436	0.04789	0.08201	0.01118	0.5155
s__Anaerococcus_sp._HMSC068A02	0.00106	0.003822	0.02637	0.09467	0.05279	0.1493	0.03096	0.5902
s__Bifidobacterium_bombi	0.008102	0.01943	0.05646	0.1575	0.0125	0.02971	0.000791	0.1591
s__unclassified_g__Bifidobacterium	0.01013	0.026	0.04465	0.06513	0.02169	0.04101	0.004895	0.355
s__Anaerococcus_prevotii	0.01701	0.0881	0.007352	0.01734	0.05126	0.09168	0.007026	0.42
s__Anaerococcus_jeddahensis	0.0005042	0.001588	0.005854	0.01667	0.06896	0.2204	0.004033	0.3179
s__Parabacteroides_distasonis	0.003652	0.01581	0.0141	0.03148	0.05683	0.1964	0.00313	0.279
s__Lactobacillus_kitasatonis	0.05346	0.1929	0.01962	0.1018	0.0006385	0.003929	0.003274	0.2872
s__Lactobacillus_kefiranofaciens	0.05775	0.09386	0.01324	0.04143	0.001235	0.004269	0.0002827	0.1025
s__Lactobacillus_taiwanensis	0.04823	0.1644	0.01529	0.04741	0.008426	0.04722	0.01485	0.5845
s__Finegoldia_sp._BIOML-A1	0.007609	0.03671	0.00657	0.01985	0.05691	0.1918	0.04319	0.5902
s__Streptococcus_milleri	0.01057	0.06028	0.05588	0.2619	0.003771	0.0151	0.02572	0.5902
s__Streptococcus_periodonticum	0.001678	0.006872	0.05501	0.2728	0.01145	0.04663	0.04447	0.5902
s__Tannerella_sp._oral_taxon_HOT-286	0.007338	0.02337	0.01805	0.0468	0.04197	0.09642	0.004303	0.3254
s__Corynebacterium_hadale	0.0125	0.05851	0.000368	0.00161	0.05315	0.2469	0.02144	0.5902
s__Peptoniphilus_sp._DNF00840	0.0006081	0.003115	0.005933	0.01444	0.0578	0.1954	0.0009813	0.1591
s__Anaerococcus_rubeinfantis	0.002648	0.00944	0.01147	0.03226	0.05019	0.1019	0.02049	0.5902
s__Peptoniphilus_vaginalis	0.002408	0.01305	0.008188	0.02516	0.05339	0.1845	0.03159	0.5902
s__Treponema_porcinum	0	0	2.706E-5	0.0001712	0.06263	0.3356	0.03663	0.5902
s__Lactobacillus_ruminis	0.03706	0.04494	0.01728	0.03501	0.007866	0.03045	0.01087	0.5054
s__Prevotella_nigrescens	0.005688	0.01907	0.01617	0.03499	0.04005	0.07888	0.005214	0.3667
s__Porphyromonas_gingivalis	0.01791	0.0588	0.01402	0.04631	0.02956	0.05436	0.0276	0.5902
s__Anaerococcus_marasmi	0.0007404	0.003583	0.008837	0.03795	0.04967	0.2103	0.003042	0.2757
s__Lactobacillus_ultunensis	0.04832	0.07949	0.008194	0.03481	0.001859	0.007662	0.01977	0.5902
s__Bacillus_sp._VMFN-A1	0	0	0.0001319	0.0008341	0.05613	0.2263	0.03923	0.5902
s__Peptoniphilus_senegalensis	0.0004427	0.002185	0.003591	0.009307	0.0489	0.1477	0.0008503	0.1591
s__Lactobacillus_psittaci	0.02044	0.03501	0.02889	0.1312	0.001131	0.004849	9.954E-6	0.02468
s__Corynebacterium_urealyticum	0.001938	0.01055	0.02515	0.06677	0.0197	0.05976	0.01853	0.5902
s__Selenomonas_sp._oral_taxon_149	1.866E-5	0.0001088	0.001761	0.00542	0.04387	0.2032	0.02468	0.5902
s__Parvimonas_sp._oral_taxon_110	0.005144	0.0277	0.01219	0.0355	0.02813	0.09126	0.02755	0.5902
s__Bacteroides_eggerthii	0.00134	0.005411	0.01061	0.03014	0.03351	0.1459	0.01191	0.5336
s__Bacillus_subtilis	0	0	0.001265	0.007362	0.04404	0.1652	0.002799	0.2581
s__Neisseria_gonorrhoeae	0.001266	0.003789	0.02006	0.0636	0.02288	0.1196	0.04084	0.5902
s__Odoribacter_splanchnicus	0.01427	0.06255	0.008131	0.02016	0.02062	0.04362	0.03889	0.5902
s__Prevotella_oris	0.005823	0.02359	0.01467	0.03932	0.02248	0.04342	0.005023	0.3595
s__Lactobacillus_pobuzihii	0.03333	0.06006	0.009291	0.0335	7.929E-5	0.000487	0.0002485	0.1025
s__Peptoanaerobacter_stomatis	0.003216	0.01503	0.005775	0.01517	0.03231	0.08743	0.01046	0.4948
s__Lactobacillus_delbrueckii	0.03155	0.04689	0.006844	0.02729	0.00284	0.01164	0.0004108	0.1397
s__Prevotella_denticola	0.004693	0.01634	0.009352	0.02336	0.02683	0.06298	0.009408	0.4652
s__Prevotella_sp._HUN102	0.003524	0.01351	0.007922	0.02339	0.02823	0.12	0.0146	0.5838
s__Peptostreptococcus_stomatis	0.0003065	0.001625	0.02478	0.1197	0.01456	0.03579	0.01444	0.5838
s__Prevotella_sp._S7_MS_2	0.004366	0.01825	0.01123	0.03117	0.02357	0.07765	0.01349	0.5599
s__Bacteroides_caccae	0.001721	0.008088	0.006714	0.01621	0.02973	0.056	0.0007956	0.1591
s__Peptoniphilus_urinimassiliensis	9.29E-5	0.0003658	0.001693	0.00391	0.03592	0.1314	0.006311	0.4132
s__Prevotella_sp._C561	0.004667	0.01574	0.01142	0.02609	0.02161	0.05422	0.0107	0.5019
s__Alloprevotella_sp._OH1205_COT-284	0.01974	0.1142	0.0133	0.07693	0.004389	0.01239	0.04764	0.5902
s__Tannerella_forsythia	0.004362	0.02012	0.01421	0.037	0.01797	0.03931	0.008018	0.4467
s__Prevotella_baroniae	0.001133	0.004642	0.006203	0.01547	0.02898	0.1581	0.04751	0.5902
s__Facklamia_hominis	0.0005077	0.001916	0.01105	0.0625	0.02448	0.06727	0.01329	0.5595
s__Treponema_socranskii	9.429E-6	5.498E-5	7.132E-5	0.0002268	0.03592	0.1759	0.04307	0.5902
s__Bacteroidales_bacterium_KA00251	0.009476	0.05247	0.005305	0.01942	0.0203	0.04695	0.04752	0.5902
s__Veillonellaceae_bacterium_DNF00626	0.0006478	0.003711	0.009225	0.02531	0.02508	0.1391	0.04419	0.5902
s__Megasphaera_sp._BV3C16-1	0.004011	0.01401	0.0266	0.1353	0.002239	0.009521	0.03445	0.5902
s__Bifidobacterium_moukalabense	0.00024	0.0007822	0.01024	0.03442	0.02186	0.09693	0.008314	0.4522
s__Bifidobacterium_sp._GSD1FS	0.00622	0.02429	0.01737	0.03452	0.008712	0.02372	0.01527	0.589
s__Anaerococcus_sp._Marseille-P3625	0.0004353	0.001767	0.002014	0.004809	0.02799	0.06619	0.001077	0.1591
s__Lactobacillus_sp.	0.02319	0.03941	0.006038	0.02392	0.001188	0.00658	0.01189	0.5336
s__Anaerococcus_sp._Marseille-P9784	0.0008553	0.003353	0.002894	0.007642	0.02638	0.06058	0.001801	0.2161
s__Prevotella_sp._DNF00663	0.004375	0.0213	0.01098	0.03079	0.01472	0.04834	0.02612	0.5902
s__Bifidobacterium_apri	0.005704	0.01345	0.01701	0.0296	0.006779	0.01788	0.02642	0.5902
s__Urinicoccus_massiliensis	0.000338	0.001287	0.002872	0.006348	0.02561	0.05678	0.006948	0.42
s__Faecalibacterium_prausnitzii	0.002714	0.008606	0.01297	0.02593	0.01232	0.02665	0.02575	0.5902
s__Peptoniphilus_lacydonensis	0.0005559	0.00303	0.003505	0.01021	0.02386	0.06235	0.002496	0.2467
s__Lawsonella_clevelandensis	0	0	0.0004414	0.002009	0.02731	0.08475	0.006381	0.4132
s__Granulicatella_sp.	0	0	0.007351	0.03947	0.01878	0.08744	0.02715	0.5902
s__Bacillus_tequilensis	0.02598	0.1476	2.752E-6	1.741E-5	2.279E-5	0.000146	0.01201	0.5336
s__Peptoniphilus_sp._BV3C26	0.001389	0.005731	0.006533	0.01744	0.01801	0.04017	0.01662	0.5902
s__Peptoniphilus_rhinitidis	0.000318	0.0007735	0.0007986	0.002566	0.02473	0.06212	0.01024	0.493
s__Corynebacterium_vitaeruminis	0	0	1.132E-5	7.161E-5	0.02574	0.1605	0.03663	0.5902
s__Bifidobacterium_pseudolongum	0.004176	0.01153	0.01634	0.03069	0.00497	0.01054	0.04228	0.5902
s__Prevotella_jejuni	0.001573	0.006946	0.0121	0.03657	0.01138	0.02388	0.003527	0.2978
s__Parabacteroides_merdae	0.001909	0.008058	0.003857	0.008801	0.01832	0.03935	0.002131	0.2318
s__Peptoniphilus_sp._oral_taxon_375	0.004031	0.01623	0.002533	0.00639	0.01746	0.04721	0.04223	0.5902
s__Peptoniphilus_raoultii	0.0008244	0.004514	0.005664	0.02095	0.01744	0.03485	0.001072	0.1591
s__Peptoniphilus_sp._HMSC075B08	0.0001602	0.000642	0.001411	0.004327	0.02235	0.06101	0.006464	0.4136
s__Lactobacillus_otakiensis	0.01471	0.02847	0.009102	0.03488	0	0	0.001112	0.1591
s__Parabacteroides_sp._D13	0.0007994	0.004201	0.002651	0.007974	0.01961	0.08895	0.01038	0.4948
s__Anaerococcus_senegalensis	0.0003811	0.001499	0.002581	0.006139	0.02005	0.07014	0.02445	0.5902
s__Bacillus_siamensis	0	0	0.0003927	0.002018	0.0224	0.06913	0.03687	0.5902
s__Facklamia_sp._HMSC062C11	0	0	0.0002103	0.0009475	0.02221	0.05778	2.794E-5	0.03799

s__uncultured_Ruminococcus_sp.	0.003283	0.01108	0.01182	0.02149	0.007048	0.01909	0.04841	0.5902
s__Prevotella_buccae	0.00367	0.01529	0.008264	0.02252	0.01012	0.02259	0.003931	0.3146
s__Actinobaculum_massiliense	2.41E-5	9.797E-5	0.01119	0.06906	0.01006	0.0334	0.01925	0.5902
s__Bifidobacterium_myosotis	0	0	0.02123	0.1152	4.693E-6	3.005E-5	0.03154	0.5902
s__Peptoniphilus_timonensis	0.0001802	0.001051	0.001619	0.005872	0.01915	0.0504	0.002665	0.2543
s__Paraprevotella_clara	0.001149	0.005243	0.008192	0.0232	0.01158	0.0341	0.01489	0.5845
s__Lactobacillus_sp._UMNPBX1	0.01516	0.02758	0.003538	0.01274	0.001673	0.008907	0.007479	0.4414
s__Bacteroides_pyogenes	0.003189	0.01223	0.009088	0.02671	0.007809	0.01588	0.03159	0.5902
s__Sphingomonas_sp._Sph1(2015)	0.01903	0.11	0	0	0	0	0.02634	0.5902
s__Bacillus_sp._FJAT-26390	0.002431	0.008884	0.01059	0.0203	0.005834	0.01837	0.02467	0.5902
s__unclassified_g__Bacteroides	0.000154	0.0006086	0.001487	0.005457	0.01701	0.04748	0.01921	0.5902
s__Phocaeicola_vulgatus	0.00156	0.007858	0.002071	0.00521	0.01479	0.06643	0.04201	0.5902
s__Peptoniphilus_sp._ING2-D1G	0.001001	0.004066	0.001883	0.004859	0.01518	0.04631	0.03134	0.5902
s__Prevotella_sp._oral_taxon_306	0.002643	0.01076	0.004603	0.01103	0.009603	0.02082	0.01626	0.5902
s__Parabacteroides_sp._20_3	0.001661	0.007776	0.003365	0.008879	0.01123	0.02215	0.006805	0.42
s__Lactobacillus_mulieris	0.01441	0.04104	0.0008216	0.005196	0.001019	0.006527	0.004367	0.3254
s__Lupinus_albus	0.002484	0.009076	0.007377	0.01249	0.006343	0.01293	0.02847	0.5902
s__Corynebacterium_sp._KPL1855	0	0	0	0	0.0161	0.103	0.02458	0.5902
s__Anaerococcus_provencensis	0.0001063	0.0005376	0.001244	0.003641	0.01465	0.04583	0.001578	0.2032
s__[Ruminococcus]_gnavus	0.002483	0.01233	0.00521	0.01663	0.008226	0.02236	0.03607	0.5902
s__Actinomyces_sp._HMSC065F12	5.119E-5	0.0002745	0.002255	0.0134	0.01349	0.07351	0.004625	0.3399
s__Syntrophomonas_zehnderi	0.0003993	0.001551	0.0006932	0.001784	0.01467	0.04919	0.02103	0.5902
s__Metaprevotella_massiliensis	0.001395	0.005669	0.005136	0.01537	0.009168	0.02011	0.03294	0.5902
s__Lactobacillus_kalixensis	0.006379	0.009092	0.008687	0.03511	0.0004395	0.002145	0.00229	0.2442
s__unclassified_g__Prevotella	0.001911	0.007916	0.004965	0.01896	0.008527	0.0255	0.04396	0.5902
s__Bifidobacterium_bifidum_CAG:234	1.059E-5	6.177E-5	0.01425	0.05237	0.0008944	0.004497	0.01679	0.5902
s__Prevotella_sp._HMSC069G02	0.001628	0.006901	0.003558	0.008114	0.009968	0.02525	0.0101	0.4905
s__Peptoniphilus_phoceensis	0.004097	0.02091	0.001002	0.002963	0.009747	0.02501	0.02395	0.5902
s__Peptoniphilus_catoniae	0.000762	0.003156	0.002422	0.007146	0.01153	0.02485	0.001095	0.1591
s__Gluconobacter_frateurii	0.002338	0.009647	0.008727	0.02024	0.003533	0.01288	0.04384	0.5902
s__Veillonella_sp._AF42-16	5.576E-5	0.0002399	0.0004836	0.001357	0.01343	0.04493	0.03599	0.5902
s__Pseudoglutamicibacter_albus	7.441E-6	4.339E-5	7.874E-5	0.000498	0.01361	0.05153	0.01935	0.5902
s__Bacteroides_sp._4_1_36	0.001471	0.006818	0.003039	0.007112	0.008916	0.01464	0.001961	0.2221
s__Corynebacterium_efficiens	0.006721	0.0346	0.005131	0.01595	0.001517	0.003308	0.006968	0.42
s__Corynebacterium_sp._HMSC069E04	0	0	6.894E-5	0.0003152	0.01315	0.06736	0.01643	0.5902
s__Prevotella_pleuritidis	0.001043	0.004395	0.006973	0.0258	0.005089	0.01092	0.0467	0.5902
s__Campylobacter_jejuni	0.0001947	0.0007907	0.005514	0.01961	0.00728	0.01829	0.001684	0.2081
s__Eggerthella_lenta	0.00138	0.005759	0.005277	0.01435	0.00533	0.01868	0.03491	0.5902
s__Bacteroides_acidifaciens	0.001196	0.006907	0.001504	0.008632	0.009066	0.02419	0.02308	0.5902
s__Peptoniphilus_obesi	3.491E-5	0.0002036	0.001222	0.003316	0.01039	0.01738	0.0001212	0.06591
s__Bacteroides_sp._HMSC073E02	0	0	3.026E-5	0.0001914	0.01148	0.0693	0.04057	0.5902
s__Lactobacillus_timonensis	0.009799	0.02071	0.001651	0.01044	0	0	8.326E-5	0.06591
s__Bacillus_sp._LYLB4	0	0	0	0	0.01141	0.0519	0.02458	0.5902
s__Parabacteroides_goldsteinii	0.0009637	0.004457	0.002681	0.006445	0.006674	0.01168	0.004315	0.3254
s__Dorea_longicatena	0.0009116	0.004701	0.002582	0.006533	0.006662	0.01603	0.02152	0.5902
s__Prevotella_micans	0.001271	0.005385	0.002303	0.005531	0.00609	0.01326	0.01556	0.5902
s__Caldalkalibacillus_thermarum	0.002056	0.006273	0.005197	0.01008	0.002371	0.008859	0.04948	0.5902
s__Prevotella_fusca	0.0003799	0.001688	0.00224	0.00501	0.006888	0.01784	0.02842	0.5902
s__uncultured_bacterium	0.001016	0.005639	0.001921	0.007233	0.006519	0.0166	0.04662	0.5902
s__Odoribacter_sp._AM16-33	0	0	0.001439	0.00829	0.007856	0.03086	0.03885	0.5902
s__Epulopiscium_sp.	0.0009109	0.003964	0.0001929	0.0007721	0.007953	0.02192	0.00254	0.2467
s__Staphylococcus_sciuri	0	0	5.124E-5	0.0003241	0.008985	0.04001	0.0008564	0.1591
s__Bacteroides_ovatus_CAG:22	0	0	6.536E-6	4.133E-5	0.008907	0.04551	0.03663	0.5902
s__Campylobacter_sp.	0	0	4.221E-5	0.000267	0.008766	0.04949	0.01494	0.5845
s__Treponema_bryantii	0	0	0	0	0.008768	0.03803	0.02458	0.5902
s__Collinsella_stercoris	0.001613	0.005416	0.005588	0.01109	0.001515	0.005337	0.02209	0.5902
s__Actinomyces_sp._S4-C9	0	0	0	0	0.008626	0.02916	0.001282	0.1788
s__Trueperella_bernardiae	0.002762	0.0161	4.681E-5	0.0002351	0.00561	0.02125	0.04994	0.5902
s__Clostridium_botulinum	0.0001474	0.0008594	0.002513	0.01051	0.005653	0.01387	0.005258	0.3667
s__Prevotella_sp._OH937_COT-195	0.001047	0.004479	0.002264	0.006619	0.004589	0.01493	0.04534	0.5902
s__Clostridium_acetireducens	0.0001222	0.0007123	0.0004163	0.001228	0.007336	0.02328	0.008252	0.4522
s__Prevotella_aurantiaca	0.0003637	0.001769	0.001087	0.003722	0.006018	0.02619	0.03916	0.5902
s__Microscilla_marina	1.284E-5	7.489E-5	0.0001002	0.0004656	0.007171	0.04063	0.04984	0.5902
s__Treponema_pectinovorum	0.0002353	0.001311	0.0008754	0.004219	0.006165	0.03275	0.03723	0.5902
s__Collinsella_aerofaciens	0.0007892	0.003218	0.004646	0.01272	0.001832	0.005794	0.03753	0.5902
s__Trueperella_pyogenes	9.277E-7	5.409E-6	0.0005477	0.003335	0.006599	0.02083	0.01755	0.5902
s__Anaerococcus_sp._Marseille-P4512	0.001424	0.005962	0.0006818	0.002253	0.004911	0.01027	0.02983	0.5902
s__Bacteroides_sp._2_1_33B	0	0	0.001505	0.006787	0.00523	0.019	0.01159	0.5297
s__Fusobacterium_pseudoperiodonticum	0.0006946	0.003873	0.001088	0.003287	0.004614	0.01817	0.03352	0.5902
s__Streptococcus_sp._HMSC034B04	0.0001222	0.0007125	0.004486	0.0177	0.001712	0.006572	0.03358	0.5902
s__Prevotella_sp._oral_taxon_317	0.002379	0.01	0.001936	0.006465	0.001903	0.004578	0.0481	0.5902
s__Prevotella_sp._HJM029	0.0004657	0.002715	0.003115	0.009253	0.002572	0.008749	0.02812	0.5902
s__Collinsella_intestinalis	0.0007443	0.003029	0.003972	0.00836	0.001157	0.004499	0.01207	0.5336
s__Allisonella_histaminiformans	3.587E-5	0.0002092	0.002537	0.007249	0.002996	0.01025	0.03169	0.5902
s__Globicatella_sp._HMSC072A10	0	0	4.936E-5	0.0003122	0.005514	0.01952	0.005908	0.3919
s__Campylobacter_rectus	0	0	0	0	0.005535	0.02819	0.02458	0.5902
s__Dialister_sp._CAG:357	0.0005222	0.001991	0.004145	0.01143	0.0008611	0.00285	0.04034	0.5902
s__Mediterraneibacter_massiliensis	0	0	0.00547	0.0339	2.371E-5	0.0001518	0.03386	0.5902
s__Bifidobacterium_thermophilum	0	0	0.001729	0.00569	0.003754	0.0199	0.042	0.5902
s__Veillonella_sp._CHU740	1.226E-5	7.149E-5	0.0002828	0.00101	0.005009	0.0175	0.02093	0.5902
s__Campylobacter_sputorum	0	0	0.0008962	0.005281	0.004379	0.01511	0.004322	0.3254
s__Campylobacter_pinnipediorum	0	0	0.0001562	0.0007783	0.004779	0.02684	0.01692	0.5902

s__Bifidobacterium_tsurumiense	0.0002133	0.0008856	0.003033	0.006774	0.001498	0.00583	0.003933	0.3146
s__Collinsella_sp._An2	0.0001086	0.0006334	0.002458	0.007671	0.001958	0.01044	0.0241	0.5902
s__Pseudomonas_sp._2588-5	0.003678	0.008794	0.000768	0.004857	0	0	0.001076	0.1591
s__Actinomyces_sp.	3.726E-5	0.0002172	5.964E-6	3.772E-5	0.004273	0.01768	0.02217	0.5902
s__Limnohabitans_sp._Rim47	0.003877	0.00868	0.0004086	0.002584	0	0	0.0002752	0.1025
s__Bacteroides_sp._3_2_5	0	0	0.0005178	0.003275	0.003419	0.01237	0.04057	0.5902
s__Veillonella_sp._3310	0	0	0.0002536	0.001274	0.003544	0.01378	0.03785	0.5902
s__Bifidobacterium_callitrichos	0	0	0.003568	0.01579	0	0	0.00779	0.4414
s__Gleimia_europaea	0	0	9.669E-5	0.0004163	0.003446	0.01437	0.005596	0.3762
s__Arcanobacterium_haemolyticum	4.477E-5	0.0002611	0.001028	0.005899	0.002397	0.008323	0.04569	0.5902
s__Leptotrichia_sp._OH3620_COT-345	0	0	0.002567	0.007392	0.0008763	0.003192	0.02464	0.5902
s__Bacteroides_sp._4_3_47FAA	0	0	0	0	0.003155	0.0127	0.009341	0.4652
s__Bifidobacterium_scardovii	0	0	0.002947	0.01651	0	0	0.02128	0.5902
s__Acidaminococcus_intestini	6.102E-5	0.0003558	0.002303	0.006704	0.0004853	0.002253	0.03885	0.5902
s__Riemerella_columbina	7.893E-5	0.0004602	0.0004503	0.001779	0.002241	0.006208	0.04676	0.5902
s__Bifidobacterium_saguini	0	0	0.002473	0.01066	0.0001209	0.0005981	0.03527	0.5902
s__Schaalia_cardiffensis	0	0	0	0	0.002399	0.01141	0.0004626	0.1398
s__Bacteroides_sp._HMSC067B03	0	0	0	0	0.002395	0.009781	0.02458	0.5902
s__Mogibacterium_sp._CM50	5.489E-5	0.0003201	0.0006921	0.001933	0.001532	0.005428	0.03042	0.5902
s__Streptococcus_sp._263_SSPC	0	0	0.0002749	0.001256	0.001972	0.008471	0.0328	0.5902
s__Akkermansia_sp._BIOML-A61	0	0	0	0	0.002233	0.01139	0.02458	0.5902
s__Actinomyces_urinae	0	0	7.025E-5	0.0004443	0.002086	0.009568	0.0155	0.5902
s__Lactobacillus_apodemi	0.001438	0.003452	0.0005276	0.002153	0.0001808	0.001013	0.02719	0.5902
s__Clostridium_butyricum	9.494E-5	0.0005481	0.0002545	0.00141	0.001793	0.00739	0.04997	0.5902
s__Prevotella_sp._PROD	1.443E-5	6.447E-5	0.000513	0.003213	0.0016	0.004504	0.02477	0.5902
s__Keratinibaculum_paraultunense	0	0	0	0	0.002067	0.01142	0.009341	0.4652
s__Pseudoscardovia_suis	0	0	0.001988	0.00833	5.271E-5	0.0003375	0.03268	0.5902
s__Leptotrichia_sp._oral_taxon_212	0	0	0.0004791	0.001988	0.001399	0.00538	0.04376	0.5902
s__Campylobacter_fetus	0	0	0.0001266	0.0008009	0.001691	0.008152	0.04057	0.5902
s__Actinomyces_culturomici	0	0	4.173E-5	0.0002267	0.001617	0.00781	0.01692	0.5902
s__Clostridium_kluyveri	0	0	7.665E-5	0.0002732	0.001545	0.005543	0.02952	0.5902
s__Parabacteroides_sp._AF17-28	0	0	0	0	0.001621	0.007707	0.009341	0.4652
s__Eubacterium_limosum	0	0	0.001107	0.004686	0.0004615	0.001534	0.0472	0.5902
s__Campylobacter_sp._RM16188	0	0	0	0	0.001451	0.005595	0.009341	0.4652
s__Bacillus_pumilus	0	0	1.475E-5	9.329E-5	0.001292	0.004834	0.03791	0.5902
s__Campylobacter_hyointestinalis	0	0	0	0	0.001303	0.006745	0.02458	0.5902
s__Propionispora_hippei	0	0	0.001226	0.007429	1.211E-5	7.754E-5	0.03386	0.5902
s__Lactobacillus_sp._395-6.2	0.001137	0.004728	9.062E-5	0.0004489	0	0	0.02807	0.5902
s__Daphnia_magna	0.000976	0.004312	0.0001886	0.001193	0	0	0.03639	0.5902
s__Parabacteroides_sp._TM07-1AC	0	0	5.907E-5	0.0002708	0.001102	0.003874	0.03687	0.5902
s__Ezakiella_peruensis	0	0	0	0	0.001136	0.004163	0.009341	0.4652
s__Campylobacter_curvus	0	0	0	0	0.001088	0.005725	0.02458	0.5902
s__Corynebacterium_atypicum	0	0	8.021E-6	3.811E-5	0.001006	0.004795	0.005602	0.3762
s__Erysipelatoclostridium_sp._An15	0	0	0.0008416	0.004999	0	0	0.02128	0.5902
s__Propionibacterium_acidifaciens	0	0	0	0	0.0008086	0.00357	0.02458	0.5902
s__Peptostreptococcaceae_bacterium_oral	0	0	0	0	0.0007752	0.003596	0.02458	0.5902
s__Butyrivibrio_sp._XPD2002	0	0	0.0007645	0.002276	0	0	0.00779	0.4414
s__Actinomyces_sp._HMSC08A01	0	0	0.0002522	0.0014	0.0005105	0.001556	0.02284	0.5902
s__Dermabacter_jinjuensis	0	0	1.153E-6	7.292E-6	0.0007039	0.00313	0.002097	0.2318
s__Bacteroides_sp._AM44-19	0	0	0	0	0.0006566	0.002249	0.02458	0.5902
s__Clostridiisalibacter_paucivorans	0	0	3.164E-5	0.0002001	0.0006154	0.003128	0.01668	0.5902
s__Aeromicrobium_sp._IC_218	0	0	0	0	0.0006296	0.003351	0.02458	0.5902
s__Dermabacter_sp._HMSC08H10	6.976E-7	4.068E-6	0	0	0.0006218	0.002396	0.03518	0.5902
s__Schaalia_suimastitidis	0	0	0	0	0.0006106	0.002417	0.02458	0.5902
s__Propioniferax_innocua	0	0	0	0	0.0005633	0.002389	0.02458	0.5902
s__Granulicatella_sp._HMSC30F09	0	0	0	0	0.0004779	0.001969	0.02458	0.5902
s__Corynebacterium_sp._zg910	0	0	0	0	0.0004622	0.002208	0.02458	0.5902
s__Helcobacillus_massiliensis	0	0	0	0	0.0004156	0.002319	0.02458	0.5902
s__Streptococcus_sp._HMSC067H01	0	0	0.0004011	0.001613	0	0	0.02128	0.5902
s__Carnobacterium_alterfunditum	0	0	0	0	0.0003666	0.002019	0.02458	0.5902
s__Ruminococcus_sp._SR1/5	0	0	0.0003193	0.001218	0	0	0.02128	0.5902
s__Lactobacillus_frumenti	0.0003073	0.001082	0	0	0	0	0.02634	0.5902
s__Pseudopropionibacterium_propionicum	0	0	0	0	0.0002791	0.001191	0.02458	0.5902
s__Corynebacterium_sp._HMSC075D04	0	0	0	0	0.0002548	0.001179	0.009341	0.4652
s__Actinomyces_bouchesdurhonensis	0	0	0	0	0.0002388	0.001137	0.02458	0.5902
s__Campylobacter_lari	0	0	0	0	0.0002129	0.0007136	0.009341	0.4652
s__Clostridium_sp._ATCC_25772	0	0	0	0	0.0001754	0.0006918	0.02458	0.5902
s__Streptomyces_capillispiralis	0	0	4.112E-5	0.0002398	0.0001305	0.0003821	0.01692	0.5902
s__Streptococcus_pantholopis	0	0	0.00015	0.0006059	0	0	0.02128	0.5902
s__Xanthobacter_sp._126	0	0	0.0001324	0.0004363	1.49E-5	9.542E-5	0.03386	0.5902

Table S5 Lefse value >2 among three groups				
Taxon	Mean	group	lda	p-value
p__Firmicutes.c__Bacilli.o__Lactobacillales.f__Lactobacillaceae.g__Lactobacillus	5.79310021	HPV	5.441834727	1.33E-06
p__Firmicutes.c__Bacilli.o__Lactobacillales.f__Lactobacillaceae	5.793103964	HPV	5.441829817	1.34E-06
p__Firmicutes.c__Bacilli.o__Lactobacillales	5.836016288	HPV	5.451754377	2.01E-06
p__Firmicutes.c__Bacilli	5.84038104	HPV	5.393110768	1.17E-05
p__Firmicutes.c__Bacilli.o__Lactobacillales.f__Lactobacillaceae.g__Lactobacillus.s__Lactobacillus_gasseri	4.378722445	HPV	4.025782465	5.82E-05
p__Firmicutes	5.853928089	HPV	5.244916965	0.000103404
p__Firmicutes.c__Bacilli.o__Lactobacillales.f__Lactobacillaceae.g__Lactobacillus.s__Lactobacillus_johnsonii	3.117370744	HPV	2.789045208	0.000113213
p__Firmicutes.c__Bacilli.o__Lactobacillales.f__Lactobacillaceae.g__Lactobacillus.s__Lactobacillus_crispatus	5.373424938	HPV	5.082623994	0.000140271
p__Fusobacteria.c__Fusobacteriia.o__Fusobacteriales.f__Leptotrichiaceae.g__Leptotrichia.s__Leptotrichia_hofstadii	2.72656131	HPV	2.333262297	0.000183355
p__Firmicutes.c__Bacilli.o__Lactobacillales.f__Lactobacillaceae.g__Lactobacillus.s__Lactobacillus_pobuzihii	2.522842071	HPV	2.259078233	0.000248487
p__Firmicutes.c__Bacilli.o__Lactobacillales.f__Lactobacillaceae.g__Lactobacillus.s__Lactobacillus_kefiranofaciens	2.761574297	HPV	2.491679336	0.000282741
p__Firmicutes.c__Bacilli.o__Bacillales.f__Bacillaceae.g__Neobacillus	3.269655959	HPV	2.843716793	0.000400665
p__Firmicutes.c__Bacilli.o__Lactobacillales.f__Lactobacillaceae.g__Lactobacillus.s__Lactobacillus_delbrueckii	2.49907836	HPV	2.20941315	0.000410818
p__Firmicutes.c__Bacilli.o__Lactobacillales.f__Lactobacillaceae.g__Lactobacillus.s__Lactobacillus_gallinarum	2.788505009	HPV	2.437767045	0.000451028
p__Firmicutes.c__Bacilli.o__Lactobacillales.f__Lactobacillaceae.g__Lactobacillus.s__Lactobacillus_paracasei	3.409423731	HPV	3.030836654	0.000645789
p__Firmicutes.c__Bacilli.o__Lactobacillales.f__Lactobacillaceae.g__Lactobacillus.s__Lactobacillus_jensenii	4.083945812	HPV	3.746970747	0.000705816
p__Firmicutes.c__Bacilli.o__Lactobacillales.f__Lactobacillaceae.g__Lactobacillus.s__Lactobacillus_acidophilus	3.774350458	HPV	3.486752226	0.000838658
p__Firmicutes.c__Bacilli.o__Bacillales.f__Bacillaceae.g__Neobacillus.s__Neobacillus_vireti	3.268293625	HPV	2.853839039	0.00100058
p__Firmicutes.c__Bacilli.o__Lactobacillales.f__Lactobacillaceae.g__Lactobacillus.s__Lactobacillus_iners	5.507663993	HPV	5.106938796	0.001095928
p__Firmicutes.c__Bacilli.o__Lactobacillales.f__Lactobacillaceae.g__Lactobacillus.s__Lactobacillus_helveticus	3.339585551	HPV	3.003059614	0.001336913
p__Fusobacteria.c__Fusobacteriia.o__Fusobacteriales.f__Leptotrichiaceae.g__Leptotrichia	2.728017514	HPV	2.279601887	0.001848025
p__Firmicutes.c__Bacilli.o__Lactobacillales.f__Lactobacillaceae.g__Lactobacillus.s__Lactobacillus_paragasseri	3.507219825	HPV	3.143039447	0.002380115
p__Firmicutes.c__Bacilli.o__Lactobacillales.f__Lactobacillaceae.g__Lactobacillus.s__Lactobacillus_kitasatonis	2.728015016	HPV	2.433544495	0.003273929
p__Firmicutes.c__Bacilli.o__Bacillales.f__Staphylococcaceae.g__Staphylococcus.s__Staphylococcus_epidermidis	3.351857144	HPV	2.805309564	0.005522171
p__Firmicutes.c__Bacilli.o__Lactobacillales.f__Lactobacillaceae.g__Lactobacillus.s__Lactobacillus_farciminis	3.396084278	HPV	2.972398857	0.009065284
p__Firmicutes.c__Bacilli.o__Bacillales.f__Bacillaceae.g__Bacillus_f__Bacillaceae.s__Bacillus_tequilensis	2.414610862	HPV	2.739491912	0.012014277
p__Proteobacteria.c__Gammaproteobacteria.o__Enterobacterales.f__Enterobacteriaceae.g__Escherichia	4.656580763	HPV	4.181309078	0.012358373
p__Firmicutes.c__Bacilli.o__Lactobacillales.f__Lactobacillaceae.g__Lactobacillus.s__Lactobacillus_amylovorus	3.220046474	HPV	2.919921996	0.013371926
p__Firmicutes.c__Bacilli.o__Lactobacillales.f__Streptococcaceae.g__Streptococcus.s__Streptococcus_sp__Z2	3.145268236	HPV	2.834128093	0.014300717
p__Proteobacteria.c__Gammaproteobacteria.o__Enterobacterales.f__Enterobacteriaceae.g__Escherichia.s__Escherichia_coli	4.64890953	HPV	4.174704005	0.014495081
p__Firmicutes.c__Bacilli.o__Lactobacillales.f__Lactobacillaceae.g__Lactobacillus.s__Lactobacillus_ultunensis	2.684141963	HPV	2.394841263	0.019773014
p__Firmicutes.c__Bacilli.o__Lactobacillales.f__Lactobacillaceae.g__Lactobacillus.s__Lactobacillus_frumenti	0.487611706	HPV	2.352239055	0.02634203
p__Proteobacteria.c__Alphaproteobacteria.o__Sphingomonadales.f__Sphingomonadaceae.g__Sphingomonas.s__Sphingomonas_sp__[illegible]	2.279378447	HPV	2.394732504	0.02634203
p__Firmicutes.c__Clostridia.o__Clostridiales.f__Ruminococcaceae.g__Faecalibacterium.s__uncultured_Faecalibacterium_sp_	2.669985431	HPV	2.173418956	0.035255909
p__Firmicutes.c__Bacilli.o__Lactobacillales.f__Streptococcaceae.g__Streptococcus.s__Streptococcus_mitis	3.876543698	HPV	3.527312147	0.047900776
p__Firmicutes.c__Bacilli.o__Lactobacillales.f__Lactobacillaceae.g__Lactobacillus.s__Lactobacillus_psittaci	2.460776356	CIN	2.141596018	9.95E-06
p__Actinobacteria.c__Actinobacteria.o__Bifidobacteriales.f__Bifidobacteriaceae.g__Bifidobacterium.s__Bifidobacterium_bombi	2.751923876	CIN	2.467549577	0.000791033
p__Firmicutes.c__Bacilli.o__Lactobacillales.f__Carnobacteriaceae.g__Trichococcus.s__Trichococcus_palustris	3.162940783	CIN	2.609879471	0.001442713

p__Actinobacteria	5.528757987	CIN	5.065402484	0.004141096
p__Actinobacteria.c__Actinobacteria.o__Bifidobacteriales.f__Bifidobacteriaceae.g__Bifidobacterium.s__unclassified_g__Bifidobacte	2.650187038	CIN	2.249952816	0.004894557
p__Actinobacteria.c__Actinobacteria.o__Bifidobacteriales.f__Bifidobacteriaceae.g__Bifidobacterium	4.770284266	CIN	4.50790815	0.00589964
p__Firmicutes.c__Bacilli.o__Lactobacillales.f__Carnobacteriaceae.g__Trichococcus	3.163450682	CIN	2.609975218	0.005934841
p__Actinobacteria.c__Actinobacteria	5.492768451	CIN	5.028392722	0.00670893
p__Actinobacteria.c__Actinobacteria.o__Bifidobacteriales.f__Bifidobacteriaceae.g__Bifidobacterium.s__Bifidobacterium_pseudocate	2.895647041	CIN	2.564371315	0.007515588
p__Proteobacteria.c__Gammaproteobacteria.o__Pseudomonadales.f__Moraxellaceae	3.251006274	CIN	2.820874198	0.008142112
p__Proteobacteria.c__Gammaproteobacteria.o__Pseudomonadales.f__Moraxellaceae.g__Acinetobacter	3.250030523	CIN	2.840217876	0.009373204
p__Actinobacteria.c__Actinobacteria.o__Bifidobacteriales	5.469743864	CIN	5.052871626	0.010099291
p__Actinobacteria.c__Actinobacteria.o__Bifidobacteriales.f__Bifidobacteriaceae	5.469743864	CIN	5.052871626	0.010099291
p__Firmicutes.c__Negativicutes.o__Acidaminococcales.f__Acidaminococcaceae.g__Phascolarctobacterium	2.931008478	CIN	2.402141656	0.01088769
p__Bacteroidetes.c__Bacteroidia.o__Bacteroidales.f__Prevotellaceae.g__Prevotella.s__Prevotella_colorans	3.080220254	CIN	2.561093999	0.012443126
p__Actinobacteria.c__Actinobacteria.o__Bifidobacteriales.f__Bifidobacteriaceae.g__Bifidobacterium.s__Bifidobacterium_longum	3.775659836	CIN	3.450496439	0.012751825
p__Actinobacteria.c__Actinobacteria.o__Bifidobacteriales.f__Bifidobacteriaceae.g__Bifidobacterium.s__Bifidobacterium_adolescenti	2.933590575	CIN	2.529069296	0.013106156
p__Firmicutes.c__Clostridia.o__Clostridiales.f__Peptostreptococcaceae.g__Peptostreptococcus.s__Peptostreptococcus_stomatis	2.394262965	CIN	2.093768645	0.014439318
p__Firmicutes.c__Negativicutes.o__Acidaminococcales.f__Acidaminococcaceae.g__Phascolarctobacterium.s__Phascolarctobacterium	2.874864449	CIN	2.317956331	0.01457404
p__Firmicutes.c__Clostridia.o__Clostridiales.f__Peptostreptococcaceae.g__Clostridioides.s__Clostridioides_difficile	2.742859901	CIN	2.336542011	0.015917911
p__Bacteroidetes.c__Bacteroidia.o__Bacteroidales.f__Prevotellaceae.g__Prevotella.s__Prevotella_amnii	3.785024894	CIN	3.514645927	0.017216551
p__Firmicutes.c__Clostridia.o__Clostridiales.f__Peptostreptococcaceae.g__Clostridioides	2.746459382	CIN	2.337827081	0.017582789
p__Actinobacteria.c__Actinobacteria.o__Corynebacteriales.f__Corynebacteriaceae.g__Corynebacterium.s__Corynebacterium_urealyti	2.400850653	CIN	2.144184606	0.018529528
p__Firmicutes.c__Bacilli.o__Lactobacillales.f__Streptococcaceae.g__Streptococcus.s__Streptococcus_pantholopis	0.17629689	CIN	2.124235375	0.021283069
p__Firmicutes.c__Erysipelotrichia.o__Erysipelotrichales.f__Erysipelotrichaceae.g__Erysipelatoclostridium.s__Erysipelatoclostridium_	0.925179081	CIN	3.055599587	0.021283069
p__Proteobacteria.c__Alphaproteobacteria.o__Rhodobacterales.f__Rhodobacteraceae.g__Roseovarius.s__Roseovarius_nitratireducens	2.784413717	CIN	2.532722401	0.027655216
p__Proteobacteria.c__Alphaproteobacteria.o__Rhizobiales.f__Xanthobacteraceae.g__Xanthobacter	0.121876769	CIN	2.324205076	0.033857123
p__Proteobacteria.c__Alphaproteobacteria.o__Rhizobiales.f__Xanthobacteraceae.g__Xanthobacter.s__Xanthobacter_sp__126	0.121876769	CIN	2.307932216	0.033857123
p__Firmicutes.c__Clostridia.o__Clostridiales.f__Lachnospiraceae.g__Mediterraneibacter.s__Mediterraneibacter_massiliensis	1.737979622	CIN	2.073864182	0.033857123
p__Firmicutes.c__Negativicutes.o__Selenomonadales.f__Sporomusaceae.g__Propionispora.s__Propionispora_hippei	1.088570157	CIN	2.366456163	0.033857123
p__Proteobacteria.c__Gammaproteobacteria.o__Pseudomonadales.f__Moraxellaceae.g__Acinetobacter.s__Acinetobacter_baumannii	3.249783495	CIN	2.715719675	0.034849928
p__Proteobacteria.c__Gammaproteobacteria.o__Enterobacterales.f__Enterobacteriaceae.g__Escherichia.s__Escherichia_fergusonii	2.722641581	CIN	2.405479598	0.041646047
p__Firmicutes.c__Clostridia.o__Clostridiales.f__Eubacteriaceae.g__Eubacterium.s__Eubacterium_limosum	1.04449104	CIN	2.045297157	0.047197771
p__Bacteroidetes.c__Bacteroidia.o__Bacteroidales	5.290220337	CC	4.936593175	1.83E-06
p__Bacteroidetes.c__Bacteroidia	5.290321435	CC	4.93663486	2.09E-06
p__Bacteroidetes	5.292753534	CC	4.937014023	3.29E-06
p__Bacteroidetes.c__Bacteroidia.o__Bacteroidales.f__Bacteroidaceae.g__Bacteroides.s__Bacteroides_uniformis	3.076827545	CC	2.71513414	9.50E-06
p__Bacteroidetes.c__Bacteroidia.o__Bacteroidales.f__Bacteroidaceae.g__Bacteroides.s__Bacteroides_ovatus	3.530541703	CC	3.233843365	1.36E-05
p__Bacteroidetes.c__Bacteroidia.o__Bacteroidales.f__Bacteroidaceae.g__Bacteroides	4.482842798	CC	4.17407709	1.60E-05
p__Bacteroidetes.c__Bacteroidia.o__Bacteroidales.f__Bacteroidaceae	4.483000484	CC	4.174188004	1.64E-05
p__Firmicutes.c__Bacilli.o__Lactobacillales.f__Aerococcaceae.g__Facklamia.s__Facklamia_sp__HMSC062C11	2.34660327	CC	2.048862776	2.79E-05
p__Bacteroidetes.c__Bacteroidia.o__Bacteroidales.f__Tannerellaceae	3.390036637	CC	3.028773244	5.03E-05
p__Bacteroidetes.c__Bacteroidia.o__Bacteroidales.f__Bacteroidaceae.g__Bacteroides.s__Bacteroides_fragilis	4.129600393	CC	3.859584767	9.10E-05
p__Bacteroidetes.c__Bacteroidia.o__Bacteroidales.f__Prevotellaceae.g__Prevotella.s__Prevotella_pallens	3.376528482	CC	3.189703375	0.000112579

p__Actinobacteria.c__Actinobacteria.o__Micrococcales	3.351299707	CC	3.008817505	0.00011752
p__Firmicutes.c__Negativicutes	4.458057257	CC	4.136957545	0.000129946
p__Firmicutes.c__Negativicutes.o__Veillonellales	4.431323459	CC	4.114202765	0.000181641
p__Firmicutes.c__Negativicutes.o__Veillonellales.f__Veillonellaceae	4.431323459	CC	4.114202765	0.000181641
p__Bacteroidetes.c__Bacteroidia.o__Bacteroidales.f__Prevotellaceae.g__Prevotella	5.061301347	CC	4.718213174	0.00019351
p__Bacteroidetes.c__Bacteroidia.o__Bacteroidales.f__Prevotellaceae	5.063418646	CC	4.715556318	0.000199783
p__Spirochaetes.c__Spirochaetia.o__Spirochaetales.f__Spirochaetaceae.g__Treponema	3.9965063	CC	3.692860338	0.000251655
p__Bacteroidetes.c__Bacteroidia.o__Bacteroidales.f__Tannerellaceae.g__Parabacteroides	3.254516287	CC	2.890479851	0.000310891
p__Proteobacteria.c__Epsilonproteobacteria.o__Campylobacterales.f__Campylobacteraceae.g__Campylobacter	3.611652875	CC	3.33235074	0.0003742
p__Proteobacteria.c__Epsilonproteobacteria.o__Campylobacterales.f__Campylobacteraceae	3.613561053	CC	3.333959071	0.0003742
p__Proteobacteria.c__Epsilonproteobacteria.o__Campylobacterales	3.621203497	CC	3.338200062	0.000500313
p__Proteobacteria.c__Epsilonproteobacteria	3.621217223	CC	3.33880422	0.000500313
p__Spirochaetes.c__Spirochaetia	4.00000624	CC	3.696362522	0.000543081
p__Spirochaetes	4.000129907	CC	3.696404874	0.000543081
p__Firmicutes.c__Negativicutes.o__Veillonellales.f__Veillonellaceae.g__Veillonella.s__Veillonella_montpellierensis	4.155511072	CC	3.910603878	0.00056476
p__Spirochaetes.c__Spirochaetia.o__Spirochaetales	3.999364595	CC	3.695593053	0.000593949
p__Spirochaetes.c__Spirochaetia.o__Spirochaetales.f__Spirochaetaceae	3.999274073	CC	3.695478801	0.000593949
p__Firmicutes.c__Bacilli.o__Bacillales.f__Bacillaceae.g__Bacillus_f__Bacillaceae	4.84853139	CC	4.497607178	0.000719243
p__Bacteroidetes.c__Bacteroidia.o__Bacteroidales.f__Bacteroidaceae.g__Bacteroides.s__Bacteroides_caccae	2.473342025	CC	2.166103028	0.000795635
p__Bacteroidetes.c__Bacteroidia.o__Bacteroidales.f__Bacteroidaceae.g__Bacteroides.s__Bacteroides_xylanisolvens	3.111280221	CC	2.766783553	0.000824292
p__Firmicutes.c__Tissierellia.o__Tissierellales.f__Peptoniphilaceae.g__Peptoniphilus.s__Peptoniphilus_senegalensis	2.68929197	CC	2.344807635	0.000850276
p__Firmicutes.c__Bacilli.o__Bacillales.f__Bacillaceae.g__Bacillus_f__Bacillaceae.s__Bacillus_velezensis	4.776811259	CC	4.421800043	0.000967216
p__Firmicutes.c__Bacilli.o__Bacillales.f__Bacillaceae.g__Bacillus_f__Bacillaceae.s__Bacillus_amyloliquefaciens	3.776003498	CC	3.394567296	0.000967216
p__Firmicutes.c__Bacilli.o__Bacillales.f__Bacillaceae.g__Bacillus_f__Bacillaceae.s__unclassified_g__Bacillus_f__Bacillaceae	3.160083734	CC	2.808950051	0.000967216
p__Firmicutes.c__Tissierellia.o__Tissierellales.f__Peptoniphilaceae.g__Peptoniphilus.s__Peptoniphilus_sp__DNF00840	2.761978784	CC	2.416928132	0.000981321
p__Firmicutes.c__Tissierellia.o__Tissierellales.f__Peptoniphilaceae.g__Anaerococcus.s__Anaerococcus_sp__Marseille_P3625	2.447048967	CC	2.141163851	0.0010769
p__Bacteroidetes.c__Bacteroidia.o__Bacteroidales.f__Porphyromonadaceae	4.616467925	CC	4.216030349	0.001265572
p__Proteobacteria	4.994968394	CC	4.3966383	0.001458075
p__Bacteroidetes.c__Bacteroidia.o__Bacteroidales.f__Prevotellaceae.g__Prevotella.s__Prevotella_intermedia	2.988633855	CC	2.607053314	0.001606206
p__Bacteroidetes.c__Bacteroidia.o__Bacteroidales.f__Tannerellaceae.g__Tannerella	2.81825727	CC	2.456832927	0.001702335
p__Firmicutes.c__Tissierellia.o__Tissierellales.f__Peptoniphilaceae.g__Anaerococcus.s__Anaerococcus_sp__Marseille_P9784	2.421220166	CC	2.114270321	0.001801063
p__Bacteroidetes.c__Bacteroidia.o__Bacteroidales.f__Prevotellaceae.g__Prevotella.s__Prevotella_bergensis	2.970749123	CC	2.563862543	0.001827712
p__Bacteroidetes.c__Bacteroidia.o__Bacteroidales.f__Bacteroidaceae.g__Bacteroides.s__Bacteroides_thetaiotaomicron	3.56780874	CC	3.225870176	0.001879822
p__Actinobacteria.c__Actinobacteria.o__Micrococcales.f__Dermabacteraceae.g__Dermabacter.s__Dermabacter_jinjuensis	0.847575973	CC	2.027272268	0.002097241
p__Firmicutes.c__Bacilli.o__Lactobacillales.f__Aerococcaceae.g__Facklamia	2.707684697	CC	2.376967788	0.002279749
p__Spirochaetes.c__Spirochaetia.o__Spirochaetales.f__Spirochaetaceae.g__Treponema.s__Treponema_denticola	3.222175012	CC	2.963886644	0.002468769
p__Firmicutes.c__Tissierellia.o__Tissierellales.f__Peptoniphilaceae.g__Anaerococcus.s__Anaerococcus_sp__Marseille_P3915	3.264460831	CC	3.033543343	0.002477413
p__Firmicutes.c__Tissierellia.o__Tissierellales.f__Peptoniphilaceae.g__Peptoniphilus.s__Peptoniphilus_lacydonensis	2.377663133	CC	2.070215572	0.002496039
p__Actinobacteria.c__Actinobacteria.o__Micrococcales.f__Microbacteriaceae	3.012995569	CC	2.666223393	0.002586862
p__Bacteroidetes.c__Bacteroidia.o__Bacteroidales.f__Bacteroidaceae.g__Bacteroides.s__Bacteroides_salyersiae	3.140218695	CC	2.944530937	0.002752031
p__Firmicutes.c__Bacilli.o__Bacillales.f__Bacillaceae.g__Bacillus_f__Bacillaceae.s__Bacillus_subtilis	2.643900473	CC	2.331530349	0.00279929

p__Firmicutes.c__Tissierellia.o__Tissierellales.f__Peptoniphilaceae.g__Anaerococcus.s__Anaerococcus_marasmi	2.696135429	CC	2.411115112	0.0030419
p__Firmicutes.c__Negativicutes.o__Veillonellales.f__Veillonellaceae.g__Dialister	3.799639074	CC	3.476342863	0.003093079
p__Bacteroidetes.c__Bacteroidia.o__Bacteroidales.f__Tannerellaceae.g__Parabacteroides.s__Parabacteroides_distasonis	2.755841256	CC	2.375843791	0.003129516
p__Firmicutes.c__Tissierellia.o__Tissierellales.f__Peptoniphilaceae.g__Anaerococcus.s__Anaerococcus_mediterraneensis	2.904329919	CC	2.600839882	0.003513982
p__Bacteroidetes.c__Bacteroidia.o__Bacteroidales.f__Prevotellaceae.g__Prevotella.s__Prevotella_bivia	4.85237585	CC	4.575214163	0.003559334
p__Bacteroidetes.c__Bacteroidia.o__Bacteroidales.f__Prevotellaceae.g__Prevotella.s__Prevotella_copri	2.720082194	CC	2.30106063	0.003639692
p__Actinobacteria.c__Actinobacteria.o__Micrococcales.f__Micrococcaceae	3.051189289	CC	2.70844319	0.003873681
p__Firmicutes.c__Tissierellia.o__Tissierellales.f__Peptoniphilaceae.g__Anaerococcus.s__Anaerococcus_jeddahensis	2.838609445	CC	2.498933747	0.004032959
p__Bacteroidetes.c__Bacteroidia.o__Bacteroidales.f__Tannerellaceae.g__Tannerella.s__Tannerella_sp__oral_taxon_HOT_286	2.623032628	CC	2.310722079	0.004302996
p__Firmicutes.c__Clostridia	4.381578429	CC	3.911697013	0.004456391
p__Firmicutes.c__Clostridia.o__Clostridiales	4.377804632	CC	3.906097328	0.004732332
p__Bacteroidetes.c__Bacteroidia.o__Bacteroidales.f__Prevotellaceae.g__Prevotella.s__Prevotella_oris	2.351846831	CC	2.001328246	0.005023301
p__Bacteroidetes.c__Bacteroidia.o__Bacteroidales.f__Prevotellaceae.g__Prevotella.s__Prevotella_nigrescens	2.602688668	CC	2.318379633	0.005214402
p__Actinobacteria.c__Actinobacteria.o__Corynebacteriales.f__Corynebacteriaceae.g__Corynebacterium.s__Corynebacterium_atypicu	1.002690575	CC	2.402659746	0.005602406
p__Firmicutes.c__Clostridia.o__Clostridiales.f__Peptostreptococcaceae.g__Peptoanaerobacter	2.561661209	CC	2.188735382	0.005883528
p__Firmicutes.c__Tissierellia.o__Tissierellales.f__Peptoniphilaceae.g__Peptoniphilus.s__Peptoniphilus_urinimassiliensis	2.555399722	CC	2.240582981	0.006311482
p__Actinobacteria.c__Actinobacteria.o__Corynebacteriales.f__unclassified_o__Corynebacteriales.g__Lawsonella.s__Lawsonella_clev	2.436312648	CC	2.252265035	0.00638126
p__Actinobacteria.c__Actinobacteria.o__Corynebacteriales.f__unclassified_o__Corynebacteriales.g__Lawsonella	2.436312648	CC	2.170820225	0.00638126
p__Actinobacteria.c__Actinobacteria.o__Corynebacteriales.f__unclassified_o__Corynebacteriales	2.436312648	CC	2.125502722	0.00638126
p__Firmicutes.c__Tissierellia.o__Tissierellales.f__Peptoniphilaceae.g__Peptoniphilus.s__Peptoniphilus_sp__HMSC075B08	2.349283304	CC	2.019976554	0.00646408
p__Bacteroidetes.c__Bacteroidia.o__Bacteroidales.f__Rikenellaceae	3.48708652	CC	3.080818153	0.006540252
p__Firmicutes.c__Tissierellia.o__Tissierellales.f__Peptoniphilaceae.g__Urinicoccus	2.459028122	CC	2.151544272	0.006540758
p__Actinobacteria.c__Actinobacteria.o__Bifidobacteriales.f__Bifidobacteriaceae.g__Bifidobacterium.s__Bifidobacterium_bifidum	2.862826053	CC	2.511230078	0.006692591
p__Bacteroidetes.c__Bacteroidia.o__Bacteroidales.f__Bacteroidaceae.g__Bacteroides.s__Bacteroides_stercoris	2.844920276	CC	2.458110277	0.006936185
p__Firmicutes.c__Tissierellia.o__Tissierellales.f__Peptoniphilaceae.g__Urinicoccus.s__Urinicoccus_massiliensis	2.408392755	CC	2.092276629	0.00694803
p__Firmicutes.c__Tissierellia.o__Tissierellales.f__Peptoniphilaceae.g__Anaerococcus.s__Anaerococcus_prevotii	2.709786935	CC	2.329527952	0.007026339
p__Bacteroidetes.c__Bacteroidia.o__Bacteroidales.f__Prevotellaceae.g__Prevotella.s__Prevotella_melaninogenica	2.582327232	CC	2.163224861	0.007631507
p__Firmicutes.c__Tissierellia.o__Tissierellales.f__Peptoniphilaceae.g__Anaerococcus.s__Anaerococcus_lactolyticus	4.174647066	CC	3.891784034	0.008049219
p__Actinobacteria.c__Actinobacteria.o__Bifidobacteriales.f__Bifidobacteriaceae.g__Bifidobacterium.s__Bifidobacterium_moukalabe	2.339714627	CC	2.014010451	0.008313742
p__Actinobacteria.c__Actinobacteria.o__Micrococcales.f__Microbacteriaceae.g__Microbacterium	3.010397425	CC	2.689988055	0.008778245
p__Bacteroidetes.c__Bacteroidia.o__Bacteroidales.f__unclassified_o__Bacteroidales	2.704422445	CC	2.234483489	0.008780572
p__Firmicutes.c__Tissierellia	5.015304442	CC	4.693508805	0.008994355
p__Firmicutes.c__Tissierellia.o__Tissierellales.f__Peptoniphilaceae.g__Anaerococcus.s__Anaerococcus_tetradius	3.874457527	CC	3.640272822	0.009163309
p__Firmicutes.c__Tissierellia.o__Tissierellales	5.014733498	CC	4.692886661	0.009187991
p__Bacteroidetes.c__Bacteroidia.o__Bacteroidales.f__Bacteroidaceae.g__Bacteroides.s__Bacteroides_sp__4_3_47FAA	1.500044858	CC	3.185835491	0.00934121
p__Firmicutes.c__Tissierellia.o__unclassified_c__Tissierellia.f__unclassified_c__Tissierellia.g__Ezakiella.s__Ezakiella_peruensis	1.055582303	CC	2.571754956	0.00934121
p__Bacteroidetes.c__Bacteroidia.o__Bacteroidales.f__Prevotellaceae.g__Prevotella.s__Prevotella_denticola	2.428744027	CC	2.069567088	0.009407946
p__Firmicutes.c__Tissierellia.o__Tissierellales.f__Peptoniphilaceae.g__Anaerococcus.s__Anaerococcus_vaginalis	3.731025699	CC	3.374327297	0.009981071
p__Proteobacteria.c__Gammaproteobacteria	4.938354711	CC	4.335028587	0.010229047
p__Firmicutes.c__Tissierellia.o__Tissierellales.f__Peptoniphilaceae.g__Peptoniphilus.s__Peptoniphilus_rhinitidis	2.393290087	CC	2.08555264	0.010241566
p__Firmicutes.c__Clostridia.o__Clostridiales.f__Peptostreptococcaceae.g__Peptoanaerobacter.s__Peptoanaerobacter_stomatis	2.50948867	CC	2.150660724	0.010462215

p__Bacteroidetes.c__Bacteroidia.o__Bacteroidales.f__Rikenellaceae.g__Alistipes	3.481820136	CC	3.048125231	0.01055504
p__Firmicutes.c__Tissierellia.o__Tissierellales.f__Peptoniphilaceae	5.012262852	CC	4.690506828	0.010696504
p__Firmicutes.c__Bacilli.o__Lactobacillales.f__Streptococcaceae.g__Streptococcus.s__Streptococcus_dysgalactiae	2.680541853	CC	2.262363382	0.011184589
p__Bacteroidetes.c__Bacteroidia.o__Bacteroidales.f__Porphyromonadaceae.g__Porphyromonas	4.61190704	CC	4.212190225	0.011479912
p__Bacteroidetes.c__Bacteroidia.o__Bacteroidales.f__Bacteroidaceae.g__Bacteroides.s__Bacteroides_eggerthii	2.525176169	CC	2.317148176	0.011907987
p__Firmicutes.c__Tissierellia.o__Tissierellales.f__Peptoniphilaceae.g__Anaerococcus	4.619757336	CC	4.320488941	0.012414601
p__Bacteroidetes.c__Bacteroidia.o__Bacteroidales.f__Odoribacteraceae	3.071202793	CC	2.808617171	0.013090671
p__Firmicutes.c__Tissierellia.o__Tissierellales.f__Peptoniphilaceae.g__Peptoniphilus.s__Peptoniphilus_sp__BV3AC2	2.868229108	CC	2.606764589	0.013186022
p__Bacteroidetes.c__Bacteroidia.o__Bacteroidales.f__Prevotellaceae.g__Prevotella.s__Prevotella_sp__HUN102	2.450827855	CC	2.115936706	0.014597789
p__Actinobacteria.c__Actinobacteria.o__Actinomycetales.f__Actinomycetaceae.g__Varibaculum.s__Varibaculum_vaginae	2.891759749	CC	2.594220242	0.015201788
p__Firmicutes.c__Tissierellia.o__Tissierellales.f__Peptoniphilaceae.g__Anaerococcus.s__Anaerococcus_hydrogenalis	3.729951783	CC	3.409027022	0.016508152
p__Actinobacteria.c__Actinobacteria.o__Streptomycetales.f__Streptomycetaceae.g__Streptomyces.s__Streptomyces_capillispiralis	0.115691075	CC	2.405614742	0.016919595
p__Fusobacteria.c__Fusobacteriia.o__Fusobacteriales.f__Fusobacteriaceae.g__Fusobacterium.s__Fusobacterium_nucleatum	3.303454786	CC	2.953180494	0.017146823
p__Firmicutes.c__Bacilli.o__Lactobacillales.f__Streptococcaceae.g__Streptococcus.s__Streptococcus_equi	2.869925821	CC	2.533670387	0.01825696
p__Bacteroidetes.c__Bacteroidia.o__Bacteroidales.f__unclassified_o__Bacteroidales.g__Phocaeicola	2.424499835	CC	2.060377816	0.020349213
p__Bacteroidetes.c__Bacteroidia.o__Bacteroidales.f__Prevotellaceae.g__Prevotella.s__Prevotella_stercorea	3.175526658	CC	2.86533079	0.0203669
p__Firmicutes.c__Tissierellia.o__Tissierellales.f__Peptoniphilaceae.g__Anaerococcus.s__Anaerococcus_rubeinfantis	2.700677274	CC	2.360284706	0.020490136
p__Actinobacteria.c__Actinobacteria.o__Corynebacteriales.f__Corynebacteriaceae.g__Corynebacterium.s__Corynebacterium_hadale	2.725475146	CC	2.309302041	0.021436601
p__Firmicutes.c__Erysipelotrichia	2.747967053	CC	2.364183729	0.021816547
p__Firmicutes.c__Erysipelotrichia.o__Erysipelotrichales	2.746373243	CC	2.351494793	0.021816547
p__Firmicutes.c__Erysipelotrichia.o__Erysipelotrichales.f__Erysipelotrichaceae	2.746373243	CC	2.356737529	0.021816547
p__Firmicutes.c__Negativicutes.o__Veillonellales.f__Veillonellaceae.g__Dialister.s__Dialister_micraerophilus	3.481863075	CC	3.121146645	0.022812992
p__Firmicutes.c__Tissierellia.o__Tissierellales.f__Peptoniphilaceae.g__Anaerococcus.s__Anaerococcus_senegalensis	2.302113752	CC	2.022016089	0.02445259
p__Actinobacteria.c__Actinobacteria.o__Propionibacteriales.f__Nocardioidaceae.g__Aeromicrobium.s__Aeromicrobium_sp__IC_218	0.799284885	CC	2.768985547	0.024578935
p__Bacteroidetes.c__Bacteroidia.o__Bacteroidales.f__Bacteroidaceae.g__Bacteroides.s__Bacteroides_sp__AM44_19	0.817376353	CC	2.799609604	0.024578935
p__Firmicutes.c__Clostridia.o__Clostridiales.f__Peptostreptococcaceae.g__unclassified_f__Peptostreptococcaceae.s__Peptostreptoco	0.889501081	CC	2.815679839	0.024578935
p__Actinobacteria.c__Actinobacteria.o__Propionibacteriales.f__Propionibacteriaceae.g__Pseudopropionibacterium.s__Pseudopropion	0.445822595	CC	2.414034672	0.024578935
p__Bacteroidetes.c__Bacteroidia.o__Bacteroidales.f__Bacteroidaceae.g__Bacteroides.s__Bacteroides_sp__HMSC067B03	1.379538722	CC	3.445628794	0.024578935
p__Proteobacteria.c__Epsilonproteobacteria.o__Campylobacterales.f__Campylobacteraceae.g__Campylobacter.s__Campylobacter_hy	1.115138494	CC	2.687110106	0.024578935
p__Proteobacteria.c__Epsilonproteobacteria.o__Campylobacterales.f__Hydrogenimonaceae	1.199041176	CC	2.305472922	0.024578935
p__Proteobacteria.c__Epsilonproteobacteria.o__Campylobacterales.f__Campylobacteraceae.g__Campylobacter.s__Campylobacter_cu	1.036899712	CC	3.284031398	0.024578935
p__Actinobacteria.c__Actinobacteria.o__Corynebacteriales.f__Corynebacteriaceae.g__Corynebacterium.s__Corynebacterium_sp__KP	2.206918136	CC	2.618872073	0.024578935
p__Proteobacteria.c__Epsilonproteobacteria.o__Campylobacterales.f__Hydrogenimonaceae.g__Hydrogenimonas	1.199041176	CC	2.877856683	0.024578935
p__Firmicutes.c__Bacilli.o__Bacillales.f__Bacillaceae.g__Gracilibacillus	0.65606889	CC	2.147098314	0.024578935
p__Verrucomicrobia.c__Verrucomicrobiae.o__Verrucomicrobiales.f__Akkermansiaceae.g__Akkermansia.s__Akkermansia_sp__BIOM	1.348900292	CC	3.661252799	0.024578935
p__Actinobacteria.c__Actinobacteria.o__Actinomycetales.f__Actinomycetaceae.g__Schaalia.s__Schaalia_suimastitidis	0.785753323	CC	2.198697697	0.024578935
p__Actinobacteria.c__Actinobacteria.o__Propionibacteriales.f__Propionibacteriaceae.g__Propionibacterium.s__Propionibacterium_ac	0.907769291	CC	2.452404757	0.024578935
p__Firmicutes.c__Bacilli.o__Lactobacillales.f__Carnobacteriaceae.g__Carnobacterium.s__Carnobacterium_alterfunditum	0.564327502	CC	2.01949118	0.024578935
p__Firmicutes.c__Negativicutes.o__Selenomonadales.f__Selenomonadaceae.g__Selenomonas.s__Selenomonas_sp__oral_taxon_149	2.642256463	CC	2.414030032	0.024677226
p__Firmicutes.c__Bacilli.o__Lactobacillales.f__Carnobacteriaceae.g__Granulicatella.s__Granulicatella_sp_	2.27378113	CC	2.048016734	0.027146275
p__Firmicutes.c__Tissierellia.o__Tissierellales.f__Peptoniphilaceae.g__Parvimonas.s__Parvimonas_sp__oral_taxon_110	2.449330282	CC	2.173471774	0.027548313

p__Actinobacteria.c__Actinobacteria.o__Bifidobacteriales.f__Bifidobacteriaceae.g__Bifidobacterium.s__Bifidobacterium_dentium	3.139274659	CC	2.831261921	0.027897213
p__Planctomycetes.c__Candidatus_Brocadiae.o__Candidatus_Brocadiales.f__Candidatus_Brocadiaceae.g__Candidatus_Kuenenia.s__	3.02440841	CC	2.718772627	0.029460533
p__Planctomycetes.c__Candidatus_Brocadiae.o__Candidatus_Brocadiales.f__Candidatus_Brocadiaceae.g__Candidatus_Kuenenia	3.02440841	CC	2.694908966	0.029460533
p__Proteobacteria.c__Gammaproteobacteria.o__Enterobacterales	4.873550623	CC	4.279104137	0.029705628
p__Planctomycetes.c__Candidatus_Brocadiae.o__Candidatus_Brocadiales.f__Candidatus_Brocadiaceae	3.024691194	CC	2.699655662	0.030113258
p__Planctomycetes.c__Candidatus_Brocadiae.o__Candidatus_Brocadiales	3.024691194	CC	2.721132721	0.030113258
p__Planctomycetes.c__Candidatus_Brocadiae	3.024691194	CC	2.719553981	0.030113258
p__Actinobacteria.c__Actinobacteria.o__Actinomycetales	4.647160487	CC	4.334476305	0.03072916
p__Actinobacteria.c__Actinobacteria.o__Actinomycetales.f__Actinomycetaceae	4.647160487	CC	4.334476305	0.03072916
p__Firmicutes.c__Tissierellia.o__Tissierellales.f__Peptoniphilaceae.g__Anaerococcus.s__Anaerococcus_sp__HMSC068A02	2.72262512	CC	2.316480579	0.030960504
p__Firmicutes.c__Tissierellia.o__Tissierellales.f__Peptoniphilaceae.g__Peptoniphilus.s__Peptoniphilus_vaginalis	2.727485223	CC	2.37891983	0.0315925
p__Proteobacteria.c__Epsilonproteobacteria.o__Campylobacterales.f__Campylobacteraceae.g__Campylobacter.s__Campylobacter_sho	3.125818583	CC	2.931270003	0.03206049
p__Planctomycetes	3.033623963	CC	2.693954237	0.032550118
p__Bacteroidetes.c__Bacteroidia.o__Bacteroidales.f__Odoribacteraceae.g__Butyricimonas	2.931388435	CC	2.744194689	0.032860025
p__Proteobacteria.c__Gammaproteobacteria.o__Enterobacterales.f__Enterobacteriaceae	4.86469542	CC	4.275495316	0.032935869
p__Proteobacteria.c__Betaproteobacteria.o__Neisseriales.f__Neisseriaceae	2.388123582	CC	2.163634796	0.033641128
p__Actinobacteria.c__Coriobacteriia.o__Eggerthellales.f__Eggerthellaceae.g__Eggerthella	2.385191596	CC	2.095940207	0.033796332
p__Proteobacteria.c__Betaproteobacteria.o__Neisseriales	2.389899542	CC	2.138052261	0.034345255
p__Actinobacteria.c__Actinobacteria.o__Corynebacteriales.f__Corynebacteriaceae.g__Corynebacterium.s__Corynebacterium_vitaerur	2.410612876	CC	2.174903888	0.036630952
p__Spirochaetes.c__Spirochaetia.o__Spirochaetales.f__Spirochaetaceae.g__Treponema.s__Treponema_porcinum	2.796946533	CC	2.487440942	0.036630952
p__Proteobacteria.c__Betaproteobacteria.o__Burkholderiales.f__Alcaligenaceae	2.395290032	CC	2.143166036	0.037698818
p__Firmicutes.c__Clostridia.o__Clostridiales.f__Peptostreptococcaceae	4.023173283	CC	3.664935644	0.038825143
p__Spirochaetes.c__Spirochaetia.o__Spirochaetales.f__Spirochaetaceae.g__Treponema.s__Treponema_medium	2.980680334	CC	2.684789891	0.039225509
p__Firmicutes.c__Bacilli.o__Bacillales.f__Bacillaceae.g__Bacillus_f__Bacillaceae.s__Bacillus_sp__VMFN_A1	2.749199419	CC	2.52577921	0.039225509
p__Actinobacteria.c__Actinobacteria.o__Actinomycetales.f__Actinomycetaceae.g__Schaalia	3.237009624	CC	2.886158327	0.040319899
p__Proteobacteria.c__Betaproteobacteria.o__Neisseriales.f__Neisseriaceae.g__Neisseria.s__Neisseria_gonorrhoeae	2.359638591	CC	2.146477665	0.040838832
p__Spirochaetes.c__Spirochaetia.o__Spirochaetales.f__Spirochaetaceae.g__Treponema.s__Treponema_socranskii	2.555508322	CC	2.366895005	0.04307408
p__Actinobacteria.c__Actinobacteria.o__Actinomycetales.f__Actinomycetaceae.g__Arcanobacterium.s__Arcanobacterium_sp__S3PF	3.265249595	CC	3.066517078	0.043355751
p__Firmicutes.c__Negativicutes.o__Veillonellales.f__Veillonellaceae.g__Dialister.s__Dialister_invisus_CAG_218	2.713235912	CC	2.408928991	0.045113276
p__Actinobacteria.c__Actinobacteria.o__Actinomycetales.f__Actinomycetaceae.g__Schaalia.s__Schaalia_turicensis	3.218457869	CC	2.859740675	0.045450037
p__Actinobacteria.c__Actinobacteria.o__Corynebacteriales.f__Corynebacteriaceae.g__Corynebacterium.s__Corynebacterium_striatum	3.207412388	CC	2.756605317	0.046807195
p__Bacteroidetes.c__Bacteroidia.o__Bacteroidales.f__Prevotellaceae.g__Prevotella.s__Prevotella_baroniae	2.46205431	CC	2.022636302	0.047513724
p__Firmicutes.c__Tissierellia.o__Tissierellales.f__Peptoniphilaceae.g__Anaerococcus.s__Anaerococcus_sp__HMSC075B03	2.826858653	CC	2.428070154	0.047860197

Table S6 Clinical baseline characteristics of patients with different HPV Oncogene status.

Characteristic		HPV Oncogene Negative (n=29)	HPV Oncogene positive (n=86)	P-value
Age (years)		47.64±2.67	52.11±1.14	0.096
BMI (Kg/m^2)		23.21±0. 70	22.50±0. 34	0.407
Post-menopause		11 (37.9)	53(61.6)	**0.032**
Smoker or passive smoker		3 (10.3)	8 (9.3)	0.869
Alcohol Drink		0 (0.0)	10 (11.62)	0.063
**Disease status**				**0.009**
	HPV group	15 (44.1)	19 (55.9)	
	CIN group	8 (20.0)	32 (80.0)	
	CC group	6 (14.6)	35 (85.4)	
RBC		4.08±0.20	3.89±0.05	0.314
RDW-SD		43.60±1.10	43.16±0.53	0.592
HB		116.64±3.83	114.38±1.91	0.848
PLT		218.80±12.42	225.42±9.26	0.830
AST		17.36±2.74	19.06±1.45	0.441
ALT		20.04±2.01	20.97±1.03	0.556
GGT		23.72±5. 48	24.75±3. 39	0.728
TBIL		12.41±0.78	12.27±0.65	0.735
DBIL		3.69±0.30	3.39±0.17	0.754
TP		66.76±1.21	66.16±0.70	0.741
ALB		41.36±0.83	41.17±0.48	0.647
UREA		4.48±0.24	4.81±0.15	0.219
CREA		54.08±1.98	53.68±1.62	0.156
UA		307.72±24.69	299.20±10.99	0.873
C1q		183.05±5.19	191.73±4.55	0.349
Glu		5.02±0.34	4.95±0.13	0.752
TC		4.23±0. 18	4.50±0. 11	0.253
TG		1.74±0. 43	1.32±0. 07	0.135
HDL-c		1.17±0.06	1.24±0.03	0.399
LDL-c		2.28±0.15	2.65±0.09	0.092
sdLDL		0.73±0.08	0.84±0.23	0.562
Shannon index		0.94±0. 12	1.15±0. 57	0.248
Chao1 index		23.22±2. 63	32.73±3. 32	**0.027**
WBC		8.12±0. 50	8.05±0. 34	0.714
Neu		6.01±0. 55	5.83±0. 34	0.942
Lym		1.62±0. 12	1.55±0. 63	0.886
Neu/Lym		4.78±0. 71	4.43±0. 36	0.782
HS-CRP		2.24±0. 57	3.56±0. 66	0.562
Estrogen		40.46±6. 68	52.84±6. 31	0.258

Note: TC total cholesterol, TG triglycerides, HDL-c high-density lipoprotein cholesterol, LDL-c low-density lipoprotein cholesterol, WBC white blood cell, Neu neutrophil, Lym Lymphocyte, HS-CRP High-Sensitivity C-reactive protein. Shannon index and Chao1 index alpha diversity of the cervical microbiome. Boldface indicated significance.
